# Light Manipulation in Organic Photovoltaics

**DOI:** 10.1002/advs.201600123

**Published:** 2016-07-06

**Authors:** Qing‐Dong Ou, Yan‐Qing Li, Jian‐Xin Tang

**Affiliations:** ^1^ Institute of Functional Nano and Soft Materials (FUNSOM)Jiangsu Key Laboratory for Carbon‐Based Functional Materials and DevicesSoochow UniversitySuzhou215123P.R. China; ^2^ Department of Materials Science and EngineeringMonash UniversityClaytonVictoria3800Australia

**Keywords:** antireflection nanostructures, light trapping, organic photovoltaics, plasmonics, polymer solar cells

## Abstract

Organic photovoltaics (OPVs) hold great promise for next‐generation photovoltaics in renewable energy because of the potential to realize low‐cost mass production via large‐area roll‐to‐roll printing technologies on flexible substrates. To achieve high‐efficiency OPVs, one key issue is to overcome the insufficient photon absorption in organic photoactive layers, since their low carrier mobility limits the film thickness for minimized charge recombination loss. To solve the inherent trade‐off between photon absorption and charge transport in OPVs, the optical manipulation of light with novel micro/nano‐structures has become an increasingly popular strategy to boost the light harvesting efficiency. In this Review, we make an attempt to capture the recent advances in this area. A survey of light trapping schemes implemented to various functional components and interfaces in OPVs is given and discussed from the viewpoint of plasmonic and photonic resonances, addressing the external antireflection coatings, substrate geometry‐induced trapping, the role of electrode design in optical enhancement, as well as optically modifying charge extraction and photoactive layers.

## 1 Introduction

Organic photovoltaic (OPV) cell is a promising technology for clean and renewable energy sources, because it may economically allow the conversion of solar power to electricity with the manufacturing of lightweight, large area, and mechanically flexible solar panels through roll‐to‐roll printing technique.^1^, ^2^, ^3^, ^4^ Starting from the pioneered bilayer heterojunction OPV,^5^ much effort has been made toward improving the power conversion efficiency (PCE) of both small molecule and polymer based cells.^6^, ^7^, ^8^, ^9^, ^10^, ^11^, ^12^ Sophisticated strategies like absorber bandgap tailoring, morphology control, and interface engineering have recently been developed, pushing the PCEs over 13% and 11% for tandem‐ and single‐junction OPVs, respectively.^3^, ^9^, ^10^, ^13^, ^14^, ^15^, ^16^, ^17^, ^18^, ^19^, ^20^ It is expected that the PCEs of OPVs will make a remarkable progress towards a competitive level of industrialization of >15%.

One challenging factor for realizing highly efficient OPVs is to overcome the low absorption efficiency of organic photoactive layers. Due to low carrier mobility and small exciton diffusion length of the commonly used small molecules and polymers in OPVs, the film thickness of the photoactive layers is normally limited to 100–200 nm for minimum charge recombination loss and efficient charge extraction. The use of ultrathin photoactive layers in OPVs inevitably leads to insufficient photon absorption and carrier generation, exhibiting low external quantum efficiency (EQE) and PCE.^21^, ^22^, ^23^, ^24^, ^25^, ^26^ To solve the trade‐off between photon absorption and charge extraction in the organic photoactive layers with an ultrathin thickness, the optical manipulation of light has become an increasingly intriguing and effective approach to boost the light harvesting efficiencies in OPVs.^18^, ^27^, ^28^, ^29^, ^30^, ^31^, ^32^, ^33^, ^34^ The introduction of periodic or random structures to the functional materials or appropriate interfaces in OPVs can redistribute the optical fields in the photoactive layers and thus trap the incident light for the enhanced photon absorption due to the internal scattering or near‐field surface plasmonic resonance effect.^35^, ^36^, ^37^, ^38^, ^39^

**Figure 1:**
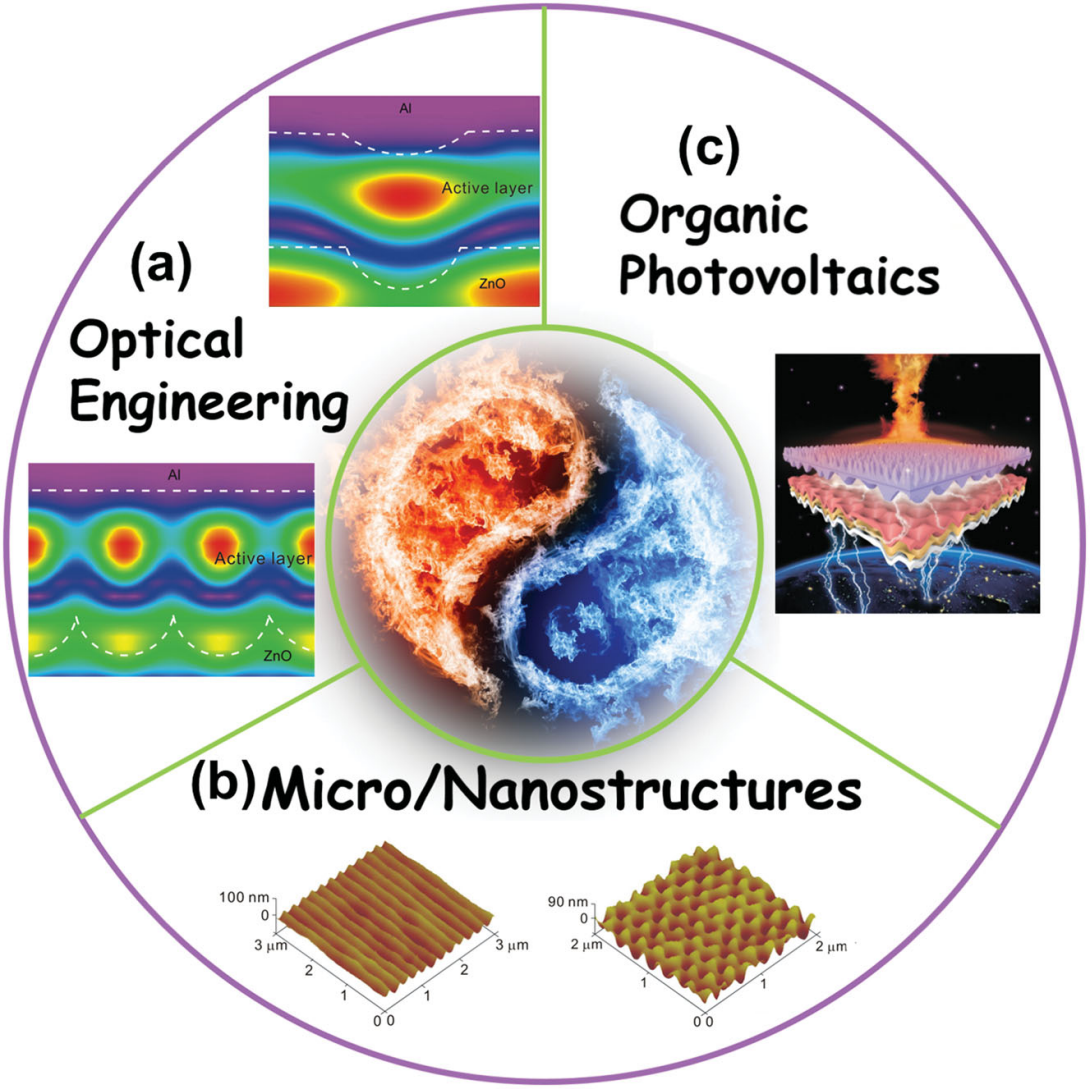
Design principle and fabrication of light trapping‐enhanced OPVs. a) Optical engineering in redistributing the optical field intensity of incident light. Reproduced with permission.^36^ b) Fabrication of micro/nano‐scale photonic structures. Reproduced with permission.^37^ c) Device integration for broadband light harvesting. Reproduced with permission.^38^

In this review we concentrate on the recent advances at the intersection of light manipulation and OPVs. A schematic envelope for light trapping‐enhanced OPVs is presented in **Figure** **1**, involving the basics of optical engineering, micro/nanostructure fabrication, and the applications in OPV panels. Section 2 of this review first focuses on the basic concepts of light manipulation using micro/nanostructures in terms of ray optics and wave optics. A series of light trapping structures implemented into various functional materials and interfaces in OPVs are then addressed. The external antireflection coatings are overviewed in Section 3. In Section 4, the light trapping induced by altering substrate geometries is discussed. Section 5 deals with the details of optical design on electrode architectures for light absorption enhancement. In Section 6, the optical modifications of charge extraction and photoactive layers are then addressed. Finally, Section 7 offers a perspective on the possible future developments of OPVs based on the strategy of light manipulation.

## 2 Design of Light Trapping Structures

To date, a variety of optical strategies have been introduced to enhance the light trapping efficiency of OPVs. From the point of view of ray optics, altering the macroscopic configurations of planar OPVs can cause the light absorption enhancement, like the folding of two cells into a V‐groove style.^40^ More popularly, microscale patterns with photonic structures are recognized as effective light trappers due to multiple bounces of incident light in the target layers. For instance, the use of microlens arrays (MLAs) provides an effective approach to increase the amount of light absorbed by the photovoltaic layers due to the decrease in the escape probability of internal rays.^41^ For the normal incidence of the solar radiation into a conventional planar photovoltaic cell, the optical path length in the photoactive layer is, for simplicity, twice the photoactive layer thickness (*t*). However, the presence of the MLA can generate an additional angular component, and the optical path length is increased to *L* = 2*t*/cosθ, where θ is the incident angle on a microlens relative to the normal direction (0° < θ < 90°).^42^ Consequently, an overall improvement in absorption efficiency (*η*_A_) can be obtained according to the Beer–Lambert law, *η*_A_ = 1 – exp(–*αL*), where *α* is the wavelength‐dependent absorption coefficient.

**Figure 2:**
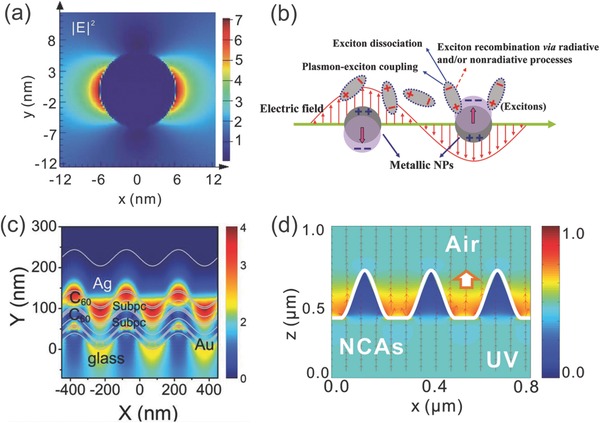
Fundamentals of optical engineering by plasmonic and scattering enhancement. a) Light trapping by exciting localized surface plasmons in metal nanoparticles. Reproduced with permission.^45^ Copyright 2012, Royal Society of Chemistry. b) Schematic of the interplay between metal nanoparticle‐induced plasmons and exciton recombination. Reproduced with permission.^46^ Copyright 2011, American Chemical Society. c) Excitation of propagating surface plasmon polaritons at the metal/organic interface in corrugated double‐junction small molecule OPVs. Reproduced with permission.^49^ d) Light management using dielectric nanostructures and calculated photon flux distribution. Reproduced with permission.^50^

To fully guide and modulate the incident light at the nanoscale for enhanced trapping and absorption in OPVs, plasmonic or biomimetic structures (e.g., moth's eye nanostructures) in term of wave optics have been implemented (**Figure** **2**), providing new pathways of broadband absorption enhancement with polarization independence and angular insensitivity.^43^, ^44^ For example, metallic nanoparticles (NPs) can be used as subwavelength plasmonic and scattering elements to couple and trap the propagating light waves into in ultrathin organic photoactive layers (Figure 2a).^45^ By virtue of the strong local field enhancement around metal NPs, increased optical absorption in organic photoactive layers can be obtained due to localized surface plasmon resonance (SPR). In this example, the field intensity around gold (Au) NPs with a diameter of 6 nm is 7‐fold enhanced at an excitation wavelength of 650 nm, indicating that the light is concentrated by the Au NP‐induced localized SPRs. As shown in Figure 2b, Wu et al. proposed the plasmon‐exciton coupling participated in charge transfer process of plasmonic‐enhanced OPVs.^46^ This photophysical process shows that the plasmonic field strongly modifies the dynamic properties of photogenerated excitons, leading to the enhanced exciton dissociation and reduced recombination of geminate excitons through radiative and/or nonradiative processes, and thus the improved photocurrent and fill factor of OPVs. On the other hand, the corrugation near a metal/semiconductor interface is favorable for the excitation of surface plasmon polaritons (SPPs), and the light in a thin absorber layer can be efficiently guided and coupled into the SPPs.^47^, ^48^ As metal contacts are standard components in OPVs, such a plasmonic coupling effect is rather attractive to benefit light absorption and harvesting. It has been demonstrated that the incorporation of periodically nanostructured metal electrodes in corrugated OPVs can support both SPPs and optical microcavity modes, and the SPP resonance can be manipulated by tuning the period of the corrugation (Figure 2c).^49^ According to the field distribution of the corrugated device with a period of 300 nm at a wavelength of 650 nm (Figure 2c), it is noteworthy that the field intensity mainly shows its maximum at the organic/silver (Ag) cathode interface with an exponential decay, resulting in ≈7% absorption enhancement of the SPP‐mediated OPV compared with the planar counterpart.

Besides the plasmonic effect with nanostructured metallic contacts, the patterning of dielectric materials in OPVs can play a crucial role in the enhancement of light harvesting in OPVs. Nanostructuring the high‐refractive‐index dielectric layers at a subwavelength scale can effectively manipulate the light absorption of OPVs due to strong optical resonances and scattering induced by the electromagnetic mode coupling in these structures.^36^ As displayed in Figure 2d, the photon flux that travels normally through a glass substrate with dielectric grooves will be diffracted and funneled into nanoscale apertures, indicating superior light coupling characteristics.^50^ Specifically, these nanoscale grooves with a period of 250 nm, groove depth of 200 nm, and duty cycle of 0.6 can effectively suppress the reflection of a flat interface by ≈7% over the nearly entire visible wavelength range, which would potentially not only benefit light in‐coupling of OPVs as external antireflective layers but also increase the internal optical scattering as photoactive components. For broadband and omnidirectional absorption enhancement, the engineering of a photonic structure is crucial because the density of optical states is proportionally related to the angle‐integrated light trapping enhancement.^51^ Consequently, the conventional material absorption limit of thin film OPVs can be overcome by means of nanophotonic light trapping schemes.

To quantificationally evaluate the photon harvest of an OPV, the optical absorption characteristics can be calculated by an 1D transfer matrix formalism (TMF). For example, Pettersson et al. modeled the photocurrent spectra of a bilayer heterojunction OPV by using the 1D TMF, which was in line with experimental results of the photocurrent and EQE spectra.^52^ Here, the photocurrent generation process can be assumed as a result of the creation and diffusion of photogenerated excitons, which are dissociated by charge transfer at the donor–acceptor interface. The optical modeling calculation using 1D TMF was also employed by He et al. to model the absorption profile in polymer bulk heterojunction (BHJ) OPVs, showing the higher absorbed incident photon flux density in an inverted structure rather than that of a regular structure.^53^ As a result, a higher short‐circuit current density (*J*_sc_) was generated in the inverted device (merely ≈5% deviation from experimental *J*_sc_), which was independent on the photoactive layer thickness.

## 3 Antireflection Coatings

When light is incident on the surface of planar transparent substrates (e.g., glass), it becomes partially reflected due to the large difference in refractive indices between air and substrates. Even for normal incidence, the light suffers from the Fresnel loss with a reflection coefficient (*R*),

(1) $$R\;={\left(\frac{n_{0}-n_{s}}{n_{0}+n_{s}}\right)}^{2}$$

where *n*_0_ and *n*_s_ are the refractive indexes of the air and substrate, respectively. For the increase in efficiency of OPVs, the use of antireflection (AR) coatings on the substrate surface is an important consideration, which can reduce the reflected light intensity and allows more light to enter the OPVs by overcoming the mismatched optical impedance at the air/substrate interface. In addition, self‐cleaning AR coatings may benefit OPV operation in real environment by blocking the accumulation of moisture and dust particles on the cell surface over time.^36^

### 3.1 Microscale Textures

**Figure 3:**
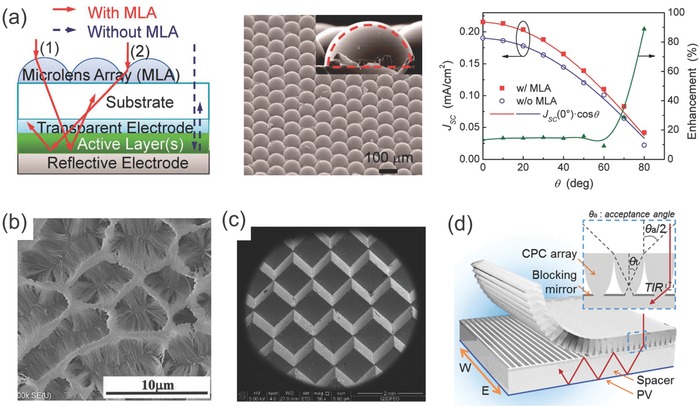
Geometric microstructures used as AR coatings. a) Schematic illustration of light transmission into OPVs with MLAs as an AR coating, scanning electron microscopy (SEM) image of a representative MLA with hemispherical shape, and its influence on device performance under angular incident light. Reproduced with permission.^42^ Copyright 2012, Royal Society of Chemistry. b) Randomly textured film of self‐aggregated alumina nanowires as an AR coating. Reproduced with permission.^57^ Copyright 2015, Royal Society of Chemistry. c) SEM image of a PDMS retroreflective texture with 1 mm‐high structures. Reproduced with permission.^58^ d) Schematic of a compound parabolic trapper (CPT) consisting of compound parabolic concentrator (CPC) array, blocking mirrors, spacer, and solar cell. Reproduced with permission.^59^

To date, various designs of microscale structures have been exploited to function as an AR coating for enhancing the light‐harvesting efficiency of OPVs. Recently, Zilio and Tvingstedt et al. incorporated the MLA as an AR coating for light trapping in OPVs, which were in conjunction with a self‐aligned array of micro apertures in a highly reflecting mirror element.^54^, ^55^ Such a system exhibited strong directional asymmetric light transmission, and an increase in cell absorption by recycling reflected photons was obtained with a photocurrent improvement of as much as 25%. Later, Myers et al. further demonstrated the effectiveness of the concept of MLAs as an AR coating for enhancing light harvesting in different OPVs (**Figure** **3**a).^42^ Due to the use of self‐assembled monolayers of polystyrene (PS) microspheres as templates, close‐packed polymer MLAs of different diameters and spacings can be easily obtained for structural parameter optimization. By directly molding such a transparent MLA on the surface of OPVs, a relative increase in the overall cell efficiency of 15–60% was achieved as a result of the reduced surface reflection and increased light path in the photoactive layer. Besides the photocurrent improvement at normal incidence, a wide‐angle enhancement (Figure 3a) was observed for OPVs with a MLA‐based AR coating, especially at large angles (>60°). This behavior is ascribed to the efficient antireflection of high‐angle incident light by the curved lens surface, indicating the capability of tackling the dynamically changed incident angle of sunlight.

Lee and co‐workers reported spectrally neutral light trapping schemes using textured plastic films attached to the substrate surface of OPVs.^56^ Randomly textured films with different levels of surface roughness (1–5 μm) can be used as potential Lambertian scattering surfaces, resulting in an enhancement in *J*_sc_ as large as 9.3% in polymer:fullerene BHJ OPVs. In addition, Kang et al. reported a high optical haze film of self‐aggregated alumina nanowire arrays to improve the light harvesting efficiency of OPVs (Figure 3b).^57^ By optimizing the etching conditions, the nanowire bundle arrays enabled an ultrahigh optical haze value up to ≈98% or high transmittance up to ≈96%. The OPV attached with such a hazy film on the glass surface exhibited an increase of the overall efficiency of 9.01% with an enhancement ratio of 10.28% due to the increased optical path length in the photoactive layer.

In contrast to the randomly textured surfaces, a periodic array of V‐groove texturing scheme was also proposed to control the incident light in a relatively controlled manner for enhancing the optical path length. Esiner et al. demonstrated a polymeric retroreflective textured sheet, which was produced separately and applied onto the glass substrate after completion of the solar cell (Figure 3c).^58^ This light trapping sheet contained an array of tilted cubic structures, several hundredths of micrometers in size. Moreover, the geometry was optimized not only to alter the angle of incident light, but also to capture the light that would be reflected out of the device. Therefore, an enhancement in PCE of 19% was quantified due to the improved light absorption and further validated by a combination of ray tracing and transfer matrix formalism modeling methods.

Most recently, Lee's group proposed a scheme combining the compound parabolic trapper with a V‐groove textured surface on a basis of contrastive analysis of light trapping capacity among MLA, V‐shaped configuration, and double parabolic trappers (Figure 3d).^59^ This light in‐coupler consisted of an 1D compound parabolic concentrator (CPC) array that concentrated the incident light, and then the transmitted light was trapped by blocking mirrors between the entrances. When further combining a metallic nanograting black electrode, the resultant PTB7‐Th based OPV yielded a PCE enhancement from 9.38% to 10.8%, validating the design of this multiscale optical system.

### 3.2 Biomimetic Nanostructures

**Figure 4:**
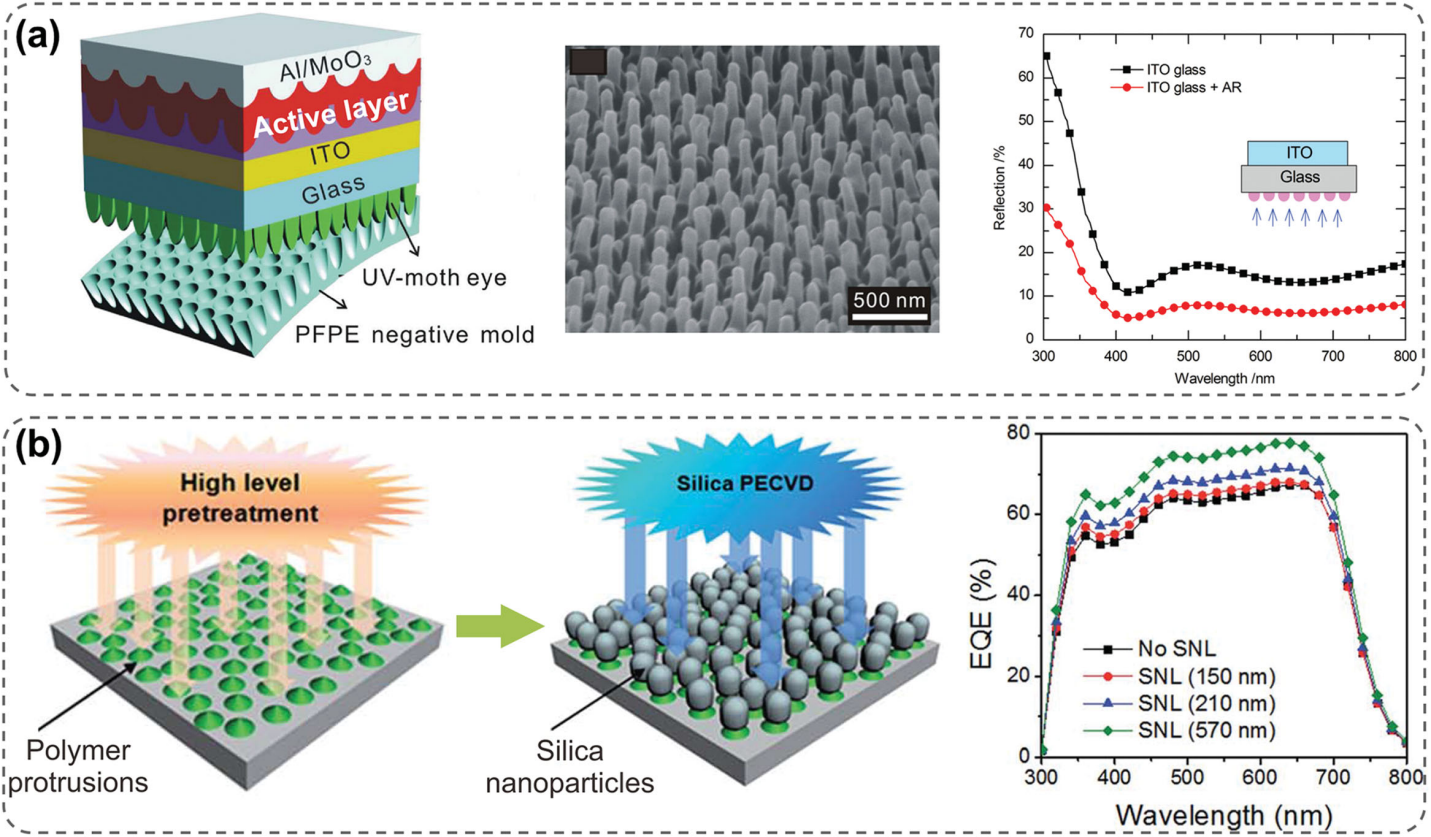
Biomimetic nanostructures for AR coatings. a) An OPV with nanoimprinted moth's eye nanostructures on UV‐curable resin on the glass surface as an AR coating, and the reflectance properties of incident light. Reproduced with permission.^36^ b) Globular silica nanoparticle arrays coated on polymer substrates using a plasma‐enhanced chemical vapor deposition (PECVD) process, and external quantum efficiency (EQE) enhancement of OPVs. Reproduced with permission.^68^ Copyright 2015, Royal Society of Chemistry.

Regardless of microscale textures, several designs of the nanostructures were conduced in OPVs as an AR coating, such as nano‐replicated periodic moth's eye structures, 1D or 2D diffraction gratings at the light incident surface.^60^, ^61^, ^62^, ^63^, ^64^ For example, Choi et al. fabricated a dual‐scale hierarchical structure as a transparent AR coating using a composite nanoimprinting mold by blending NPs into the ultraviolet (UV)‐curable resin for large area OPVs.^63^ Recently, Chen et al. developed a self‐cleaning AR coating featuring biomimetic moth's eye nanostructures in quasi‐periodically arranged gradient shape through soft nanoimprint lithography (NIL) (**Figure** **4**a).^36^ The scanning electron microscopy (SEM) image of an imprinted UV‐curable resin layer on glass surface shows a uniform array of convex nanostructures with a period of 200 nm and a groove depth of 180 nm. As displayed in Figure 4a, the integration of a moth' eye nanostructured AR coating onto indium‐tin‐oxide (ITO) glass substrate resulted in a broadband reduction of ≈7% in the average reflectance at normal incidence. Additionally, such a moth's eye AR coating exhibited self‐cleaning characteristic with high water contact angles (≈134°). When integrating a moth's eye AR texture along with the moth's eye nanostructured photo active layer, the OPV device based on a poly(3‐hexylthiophene‐2,5‐diyl):indene‐C60 bis‐adduct (P3HT:ICBA) BHJ yields a *J*_sc_ enhanced by 24.3% and a promising PCE of 7.86%.^36^ With the use of a more efficient absorber of thieno[3,4‐b]thiophene/benzodithiophene:[6,6]‐phenyl C71‐butyric acid methyl ester (PTB7:PC_71_BM), the light harvesting efficiency of OPVs with the similar dual‐side biomimetic moth's eye nanostructures was further increased, yielding an enhanced PCE of 9.33% without sacrificing the charge transport properties.^65^

Hyun et al. proposed to directly attach a soft elastomeric stamp with a 2D hexagonal array of nanopillars onto the glass substrate to enhance light in‐coupling for OPVs.^66^ Driven by 3D interference of diffracted orders, the nanopillars served as phase‐shift elements that scattered light through the encapsulating layer and transparent electrode into the photoactive layer. Similarly, wavelength‐scale inverted pyramid structures with low reflectance and excellent low haze were fabricated on the back surface of glass using a soft lithographic technique with etched GaN molds.^67^ Compared to the flat device, an enhancement of 18% of the PTB7 based OPV with a wavelength‐scale structured haze film was achieved, yielding a PCE of 8.41%.

Recently, silica (SiO_2_) nanomaterials have attracted great interest in fabricating large‐area, low‐cost AR films. For example, Yun and co‐workers produced the SiO_2_ NPs‐based light scattering layer on the substrate surface in bendable OPVs with enhanced light absorption (Figure 4b).^68^ A closely packed arrangement of nanoscale polymer protrusions was firstly formed on the surface of a highly flexible and heat‐sensitive poly(ethylene terephthalate) (PET) substrate by controlling the pretreatment time. A nearly quasi‐periodic array of discrete SiO_2_ NPs was subsequently self‐assembled on the plasma‐treated PET surface at room temperature via plasma‐enhanced chemical vapor deposition (PECVD) technique with controlled deposition thickness. By optimizing the dimensional parameter of SiO_2_ NPs, the flexible OPV yielded a PCE of 7.4% with an improvement ratio of 13% compared to the reference device without a SiO_2_ NP layer. Alternatively, a nanostructured AR coating could be simply fabricated on the back surface of an ITO‐coated glass substrate by self‐assembling a monolayer of SiO_2_ nanospheres with the immersion into the negatively charged monodispersed nano‐SiO_2_ colloidal solution (≈126 nm in diameter).^69^

For top‐illuminated OPVs, a transparent metal‐dielectric electrode is typically used as the top electrode.^70^, ^71^ For optically improving the light in‐coupling efficiency, Ham et al. proposed a dielectric/metal/polymer stack as a top transparent electrode, in which polydimethylsiloxane (PDMS) was involved as the polymer layer due to its small complex refractive index.^70^ When well‐ordered nanopatterns were implemented on the PDMS layer, this nanocomposite electrode was capable of reducing the reflection loss at the air‐top electrode interface, and the transmittance was insensitive to the polymer thickness. The resulting OPV with such a stack exhibited the enhanced light absorption with the improved PCE from 4.46% to 6.75%. On the other hand, Choy and co‐workers designed a hybrid optical nanostructure top electrode for semitransparent OPVs, which was composed of an ultrathin Ag film for charge collection, the embedded high‐index Si nanoparticles (NPs) as the low‐loss scatter, and the top index‐matching layer of tris(8‐hydroxyquinolinato) aluminum (Alq_3_).^71^ Such a metal/nanoparticle/dielectric stack could increase the light in‐coupling efficiency in long wavelength region due to the scattering of Si NPs, as well as in short wavelength region due to the Alq_3_ layer with additional synergetic improvement in this hybrid electrode nanostructure. The corresponding PCE for top‐illuminated OPVs was enhanced by ≈34%, with an efficiency recovery up to 68%.

Taking advantage of conversion and manipulation of light via luminescent down‐shifting (LDS), Nam et al. developed a multifunctional transparent and luminescent LDS platform bearing sub‐wavelength nanopatterns in OPVs.^72^ The nanostructures that were designed to enhance both absorption and emission profiles while maintaining visible transparency, were tailored to OPVs to accommodate an improved spectral response to the incident light. The distinctively enhanced efficiency and lifetime of OPV devices were mainly attributed to the combined effects of nanopattern‐derived AR and LDS properties.

Regardless of the superior AR capabilities from both microscale textures and biomimetic nanostructures, the following factors should be taken into account. First, different mechanisms are invovled for the AR process when using microscale textures or biomimetic nanostructures. The ray‐optical schemes using microtextures lead to multiple reflection of incident light on the organic photoactive layers, whereas the biomimetic nanostructures utilize the wave‐optical property of light to interact with the subwavelength structures. Second, the microscale AR coatings generally have the advantages of intuitive geometries, broadband response, and manufacture scalability. However, they are still limited by narrow angular tolerance and reflection loss induced by multiplied optical bounces. In contrast, the biomimetic AR coatings such as moth's eye nanostructures can simultaneously achieve broadband and omnidirectional anti‐reflectivity with polarization insensitivity in spite of the stringent specification of nanoscale structural design and fabrication. Besides inherent superiorities in thin form factor and self‐cleaning characteristics, these biomimetic nanostructures offer a great promise in producing large scale OPV panels at low cost through soft NIL (e.g., polymer‐based) and solution processing (e.g., SiO_2_ NP‐based) techniques.

## 4 Substrate Geometry‐Induced Trapping

**Figure 5:**
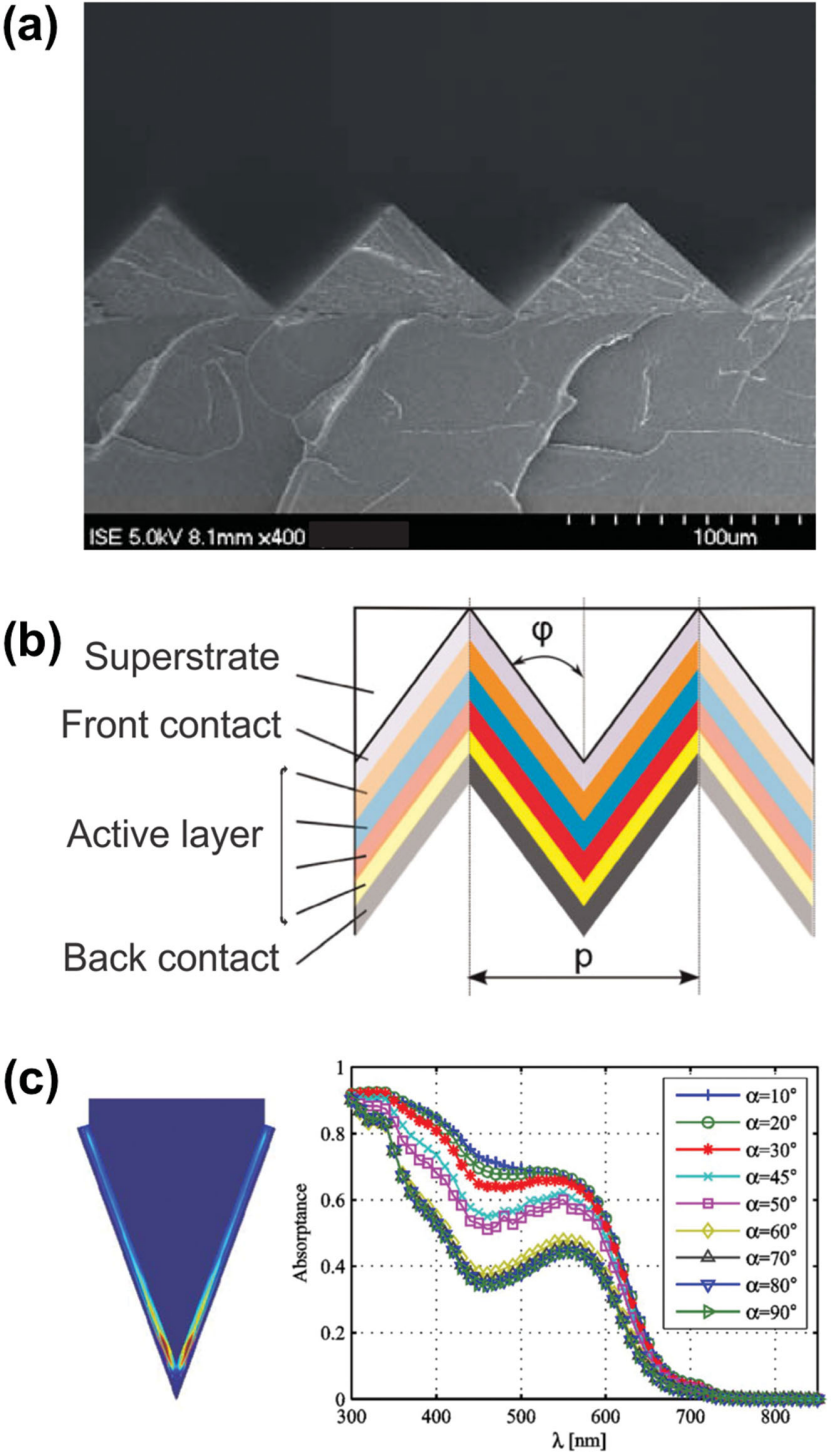
Substrate geometry‐based light trapping. a) Cross‐section SEM image of a microprism structure with a period over 100 μm. Reproduced with permission.^21^ b) Schematic of a folded OPV on a V‐groove. Reproduced with permission.^74^ c) Simulated energy dissipation at a folding angle of 20° (left) and absorption evolution with increasing folded angle (right). Reproduced with permission.^75^ Copyright 2008, American Institute of Physics.

To date, most of the state‐of‐the‐art efficiencies of OPVs have been accomplished with a flat substrate geometry. Niggemann and co‐workers pioneered the use of a close‐to‐macroscale V‐grooved microprism substrate for a folded OPV, which was regarded as a new cell geometry in contrast to the most widely used planar device architecture (**Figure** **5**a).^21^ These folded solar cell architectures benefit from the illumination under inclined incident angles and multiple reflections.^73^ Figure 5b shows the cell structure of a typical V‐grooved OPV, which exhibited substantially improved photocurrent over wide incident angles as compared to the planar cell.^74^ Taking into account the interference, refraction, and reflection effects, the simulated energy dissipation and absorption evolution in a V‐shaped cell clearly reveal the effect of multiple reflections with larger energy dissipation close to the groove bottom and larger absorption for smaller folding angles (Figure 5c).^75^ As a geometrical alternative for thin film solar cells, Inganäs et al. proposed a folded reflective tandem OPV by folding two flat yet spectrally different cells toward each other, which provided a way to obtaining broadened absorption spectra and enhanced light trapping with an enhancement factor of ≈1.8 in PCE.^40^

Besides the V‐grooved cell architecture, other substrate geometries were investigated for enhancing the light trapping. One alternative optical geometry for OPVs is fiber‐based. As demonstrated by Carroll et al.,^76^, ^77^ OPVs on standard multimode optical fibers exhibited excellent light harvesting performance due to the formation of confined radiation modes. The absorption of the photoactive layers was dominated by the evanescent coupling of the light at small incident angles and far‐field scattering of the light from the fiber at higher angles. A fiber‐shaped OPV cell utilizing concentric thin films of small molecular organic compounds was demonstrated by O'Connor et al. with the fiber device efficiency nearly independent of illumination angle.^78^ Numerical models using ray tracing and optical path iteration were correspondingly proposed for the simulation of light transmission, absorption, and loss in fiber‐based OPVs.^79^, ^80^ Lee et al. reported a coaxial wire system for flexible OPVs with a PCE over 3%, where a thin metal wire (the primary electrode) coated with a phase‐separated polymer photoactive layer was wrapped around a second wire (the counter electrode) coated with the Ag film.^81^ Inspired by the fiber architecture, Carroll's group also demonstrated a waveguiding tube‐based optical geometry to improve the OPV performance.^82^ By fabricating polymer‐based OPVs onto thin tubular light pipes, the optical energy could be effectively captured within the photo­active layer without reflective losses at the front and rear surfaces of the devices as compared to the typical planar structures. Similarly, transference cylindrical substrate was also involved for the light trapping structure to achieve high efficiency OPVs, showing the calculated 200% enhancement ratio of light absor­ption as compared to the case of a flat substrate.^83^ Tvingstedt et al. demonstrated efficient light trapping by combining echelle grating structures with semitransparent electrodes in the ray domain approach, in which the large tilted reflective structures of a ladder type “echelle” or blazed grating with a pitch of 55 μm and a height of 40 μm could shift the propagation direction of light into angles of total internal reflection.^84^

## 5 Electrode Engineering

On the one hand, transparent front electrodes, which simultaneously conduct electrical current and transmit light mostly in the entire visible spectral range, are of paramount importance for the OPV technology. Thin metal films or conductive oxide layers are widely used in OPVs as transparent front electrodes due to their excellent electrical conductivity and suitable optical transmittance. On the other hand, opaque metal films with high reflectivity are the most popular and feasible choice as rear electrodes and back mirrors. For efficient light transmission and trapping, the optical properties of both front and rear electrodes have been intensively engineered with various photonic structures, thereby enhancing the light harvesting in the sandwiched photoactive layers.^85^, ^86^

### 5.1 Transparent Front Electrodes

#### 5.1.1 Diffractive Oxide Electrodes

**Figure 6:**
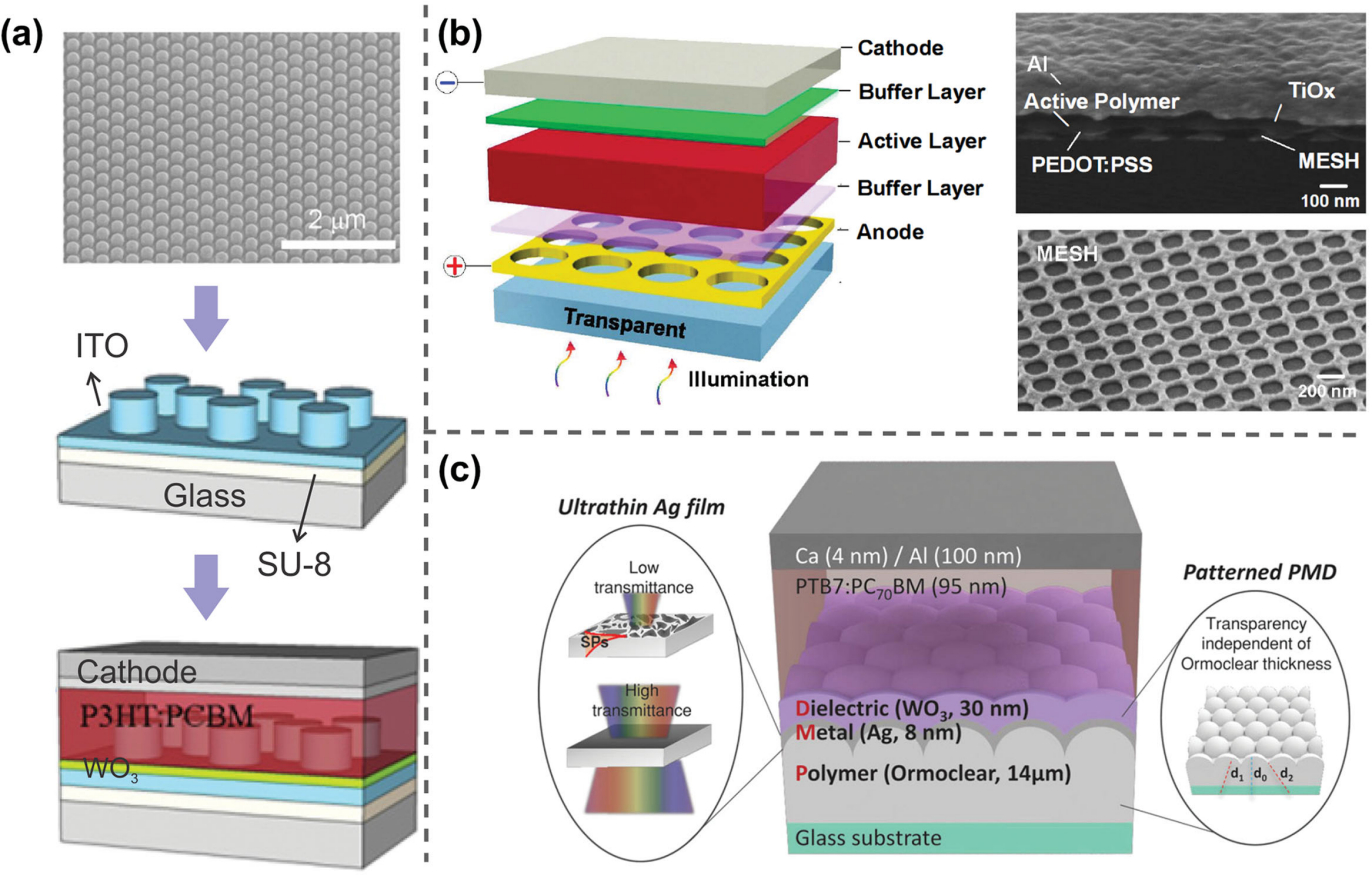
Enhanced light harvesting via optical engineering of transparent front electrodes. a) Schematic of the fabrication of nanostructured transparent front electrode by depositing ITO film on top of nanoimprinted photoresist. Reproduced with permission.^87^ Copyright 2013, IOP Publishing. b) Plasmonic cavity with subwavelength hole‐array metal front electrode. Reproduced with permission.^94^ Copyright 2013, Optical Society of America. c) Schematic of a nanopatterned polymer/metal/dielectric (PMD) film as the transparent front electrode with a smooth ultrathin Ag layer and low‐refractive‐index Ormoclear layer. Reproduced with permission.^101^

The preliminary modification of the transparent front electrodes on rigid or flexible substrates may easily adapt to most OPV systems with negligible influence on the subsequent device processing. To improve the light in‐coupling efficiency of the traditional ITO front electrode, Lopez et al. fabricated a nanopatterned transparent electrode by depositing ITO onto a patterned substrate for OPVs (**Figure** **6**a).^87^ A commonly used negative transparent photoresist SU‐8 was firstly patterned to form a 2D hexagonal nanoscale array of subwavelength posts. An ITO film was then deposited on top of SU‐8 by pulsed laser deposition to form an optically continuous nanostructured conductive electrode. Light trapping and electrical characteristics were synergistically enhanced in such a nanostructured OPV. Similarly, efficiency‐enhanced flexible OPVs were achieved by using a nanopatterned indium zinc oxide anode.^88^

Apart from the fabrication of transparent conductive oxide film on top of nanostructured substrates, directly texturing ITO film was employed for the light in‐coupling enhancement in OPVs. Xu and co‐workers produced the textured ITO front electrode by wet‐chemical etching with PS nanospheres as the etching template.^89^ The OPVs on this textured ITO electrode showed an enhancement ratio of 15% in efficiency due to the morphological and interfacial modifications as well as enhanced light absorption. Instead, Kim et al. fabricated an ITO nanohelix array by an oblique‐angle‐deposition technique to function as an effective AR coating as well as a light scattering layer, which can significantly enhance the light harvesting in OPVs.^90^

In addition, theoretical studies are well complementary to experimental demonstrations, and of great benefit to the design and optimization of nanostructuring the transparent front electrodes in OPVs. Based on numerical simulation Fan et al. predicted that a 8–15% increase in photocurrent can be achieved by introducing 1D, 2D and multilevel wavelength‐scale ITO grating structures in an OPV stack, which arises from the significant absorption resonances.^91^ Moreover, Peres et al. theoretically showed that inserting a photonic crystal (PC) with a period of ≈1200 nm into the ITO electrode caused the optimized optical resonance, resulting in the enhanced light trapping and a 23% increase in the absorption of OPVs.^92^

#### 5.1.2 Ultrathin Metal Electrodes

As a promising alternative to conductive oxide electrode, thin metal films with high electrical conductivity have gained increasing popularity in OPVs as transparent front electrodes because of the introduction of SPR or microcavity effect. In this subsection, we will focus on the aspect of the SPR‐active metal film. The microcavity effect induced by transparent metal front electrodes will be discussed in details in Section 5.3.2.

Reilly et al. explored the plasmonic Ag films as a stand‐alone front electrode in OPVs, in which the Ag films were fabricated at sub‐monolayer coverage with randomly perforated nanoholes by using colloidal lithography techniques and metal vapor deposition.^93^ The nanostructured Ag electrode was favorable to the SPR‐enhanced photo‐conversion, surpassing the efficiency of the ITO device. After this pioneering work, various concepts using plasmonic metal electrodes with 1D and 2D photonic structures were implemented in OPVs, validating the effectiveness of enhanced light harvesting with the replacement of ITO or planar metal film electrode.^94^, ^95^, ^96^, ^97^ A representative gold (Au) nanomesh electrode with a subwavelength hole array templated by PS nanospheres is shown in Figure 6b, which was helpful to improve light trapping of OPVs with the plasmonic cavity effect.^94^ An ultrathin OPV stacked on this Au nanomesh exhibited an average light in‐coupling efficiency of 90% with broadband and omni acceptance.^94^, ^95^

Min et al. performed a theoretical study on the partial substitution of metal thin‐film electrode by periodic metallic gratings, showing the broadband optical absorption enhancement for transverse magnetic (TM)‐polarized light due to the large plasmonic field enhancement in the vicinity of the grating strips.^98^ Guo and co‐workers experimentally demonstrated the efficiency enhancement of OPVs using 1D Ag nanograting electrode (strip width ≈55 nm) due to the SPR and waveguide effects, and the optimized PCE was enhanced by about 35% compared to that of the ITO device under unpolarized light illumination.^99^ Further enhancement in light harvesting efficiency can be expected by tuning the period of the Ag nanogratings to match the SPR‐enhanced spectral range with the absorption peak of the photoactive layer. Instead of using shadow masks, Polman et al. realized the 2D polarization‐independent Ag nanowire networks through large‐area soft NIL, which can function as transparent front electrode and a light trapping structure with minimized reflection and parasitic absorption loss.^100^

Another style of plasmonic metal electrodes used in OPVs is depicted in Figure 6c,^101^ which are formed by depositing a continuous metal thin film on a nanopatterned substrate. Recently, Ham et al. realized a highly transparent conducting polymer/metal/dielectric stack integrated with well‐ordered 2D convex nanodome patterns, which was successfully applied as the front electrode in OPVs with increased photocurrent as compared to the use of ITO electrode.^101^

### 5.2 Reflective Rear Electrodes

#### 5.2.1 Distributed Bragg Reflectors

1D PCs or dielectric multilayers are known as a distributed Bragg reflector (DBR), providing a way to manipulate the photon propagation. Photons with energies lying in the photonic band gap cannot propagate through the DBR.^102^ The tunability of the reflection properties of DBRs is highly attractive by controlling the alternate thickness and refraction index of multilayers. Therefore, a series of DBR configurations were explored for fabrication of highly efficient semitransparent OPVs based on various absorbers.^103^, ^104^, ^105^, ^106^

**Figure 7:**
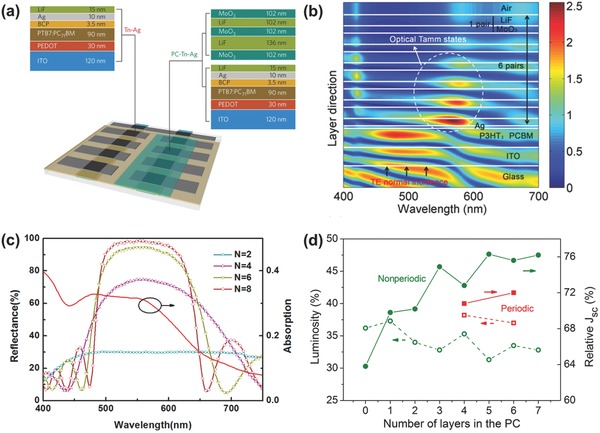
Distributed Bragg reflectors (DBRs). a) A transparent OPV employing a multilayered light‐trapping architecture. Reproduced with permission.^32^ Copyright 2013, Nature Publishing Group. b) Electric field distribution in OPV with a periodic MoO_3_/LiF DBR. Reproduced with permission.^107^ Copyright 2013, American Chemical Society. c) Optical properties of multilayered WO_3_/LiF DBRs and absorption spectrum of the absorber. Reproduced with permission.^108^ Copyright 2014, American Chemical Society. d) Luminosity and *J*_sc_ comparison of OPVs as a function of the number of layers between nonperiodic multilayer and optimal periodic DBRs. Reproduced with permission.^109^ Copyright 2015, SPIE.

To enhance the trapping of the electromagnetic field in semitransparent OPVs over a wide wavelength range, 1D photonic structures are usually integrated above the rear semitransparent electrode. As shown in **Figure** **7**a, a layered DBR was proposed and implemented in semitransparent OPVs, which resulted in the light harvesting recovery up to almost 80% that of its opaque counterpart by promoting the photon‐to‐charge conversion.^32^ As a result, the semitransparent OPV based on a PTB7:PC_71_BM photoactive layer exhibited a PCE of 5.6% as well as 30% visible light transmission. In addition, modifying the layer structure can tune the device color without significantly altering cell performance.

According to the theoretical mapping of the electric field distribution in semitransparent OPVs with a DBR, the light trapping effect can be evaluated. In the case of molybdenum trioxide (MoO_3_)/lithium fluoride (LiF) DBR system (Figure 7b), the field intensity in the photoactive layer was remarkably enhanced in comparison with that of the reference device without DBR, and an optical Tamm state formed in the DBR was predictably excited by the free space incidence, which was favorable for optical trapping.^107^ In addition, the optical properties of 1D DBRs are highly dependent on their pair number. As observed in Figure 7c of a multilayered tungsten trioxide (WO_3_)/LiF,^108^ the reflectance of 1D DBRs at a specific wavelength range can be gradually enhanced with increasing pair number. Therefore, the light trapping capability of these DBR geometries can be tuned with respect to the variation of the number of layers and the periodicity in the DBR. As indicated by Martorell et al. (Figure 7d),^32^, ^109^ the photocurrent of a PTB7‐based OPV increased rapidly with respect to the increase of layer numbers in the DBR but saturated beyond five layers. More interestingly, non‐periodic DBRs used to trap near‐infrared and near‐ultraviolet photons exhibited relatively better performance compared to their periodic counterparts, which was attributed to the optimal interference at each wavelength for largest efficiency while at the same time maintaining a good transparency in most the visible wavelengths.

In addition to the use of DBRs for rear metal electrodes, light trapping can be realized by modifying the transparent front electrodes by virtue of the DBR concept.^110^ Sun and co‐workers presented a theoretical insight to the 1D DBR structure that broadband absorption enhancement is related to the excitation of optical Tamm states.^111^ Pastorelli et al. experimentally demonstrated the enhanced light harvesting through sandwiching 1D PCs of titanium oxide (TiO_2_) and SiO_2_ units between ultrathin metal transparent electrode and glass substrate.^112^ Upon the optimum layer configuration of such a nonperiodic PC, semitransparent OPV device exhibited a PCE of 5.3%, corresponding to 90% of the opaque cell, while possessing 21% visible transparency.

#### 5.2.2 Plasmonic Electrodes

In the past few years, the emerging field of plasmonics has received extensive explorations in OPVs for light trapping enhancement at the nanoscale, well below the scale of the wavelength of light in free space.^24^, ^25^, ^28^ Especially, plasmonic nanostructures of metallic rear electrodes have exerted great influence on redistributing optical field and scattering light waves inside a cell. Accordingly, various plasmonic electrodes (e.g., metallic gratings) were designed to improve absorption in OPVs along with different implementation strategies such as NIL.

**Figure 8:**
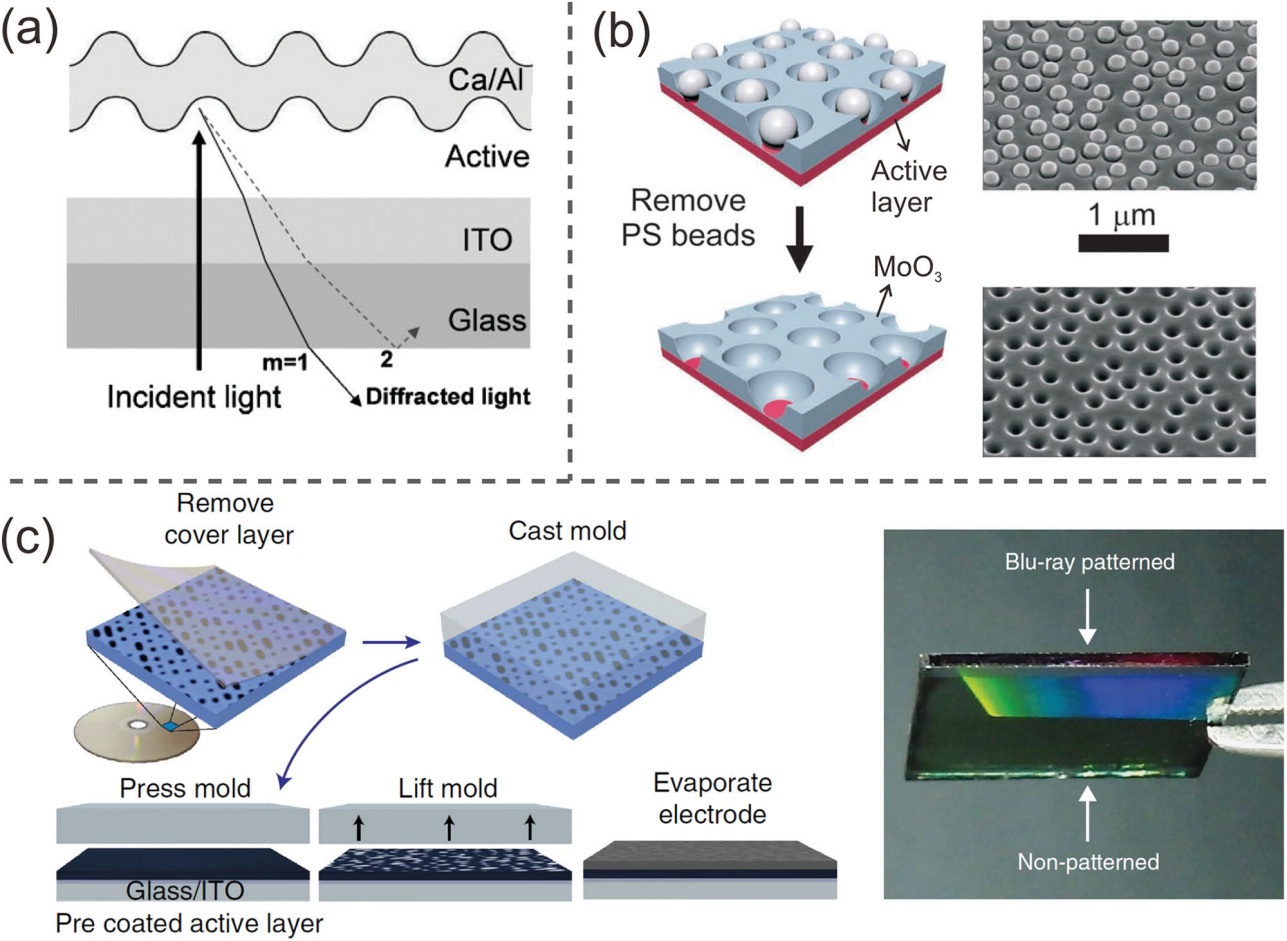
Plasmonic nanostructures at metallic rear electrodes. a) Schematic of an OPV with periodic surface relief gratings. Reproduced with permission.^113^ b) Nanostructured metal rear electrode through polystyrene (PS) nanosphere template. Reproduced with permission.^125^ c) Schematic diagramming the process and optical images of solar cells via Blu‐ray movie discs as quasi‐random nanoimprinting templates. Reproduced with permission.^130^ Copyright 2014, Nature Publishing Group.

Commonly, SPPs in the style of propagating light waves along the interface between a metal and a semiconductor material can be excited in OPVs by employing a periodically nanopatterned metal film, resulting in near field enhancement and improved optical absorption. The OPV structure with metallic gratings at the rear electrodes represents the typical light trapping scheme by the excitation of SPPs (**Figure** **8**a).^113^ Various methods utilizing 1D or 2D periodic gratings were investigated for SPP‐mediated light absorption at tunable wavelengths by tuning the periodicities and duty cycles. You et al. demonstrated one representative utilization of SPPs in polymer‐based OPVs by stamping 1D nanograting onto the photoactive layer for the formation of plasmonic metal grating rear electrode, causing an increase in PCE from 7.20% to 7.73%.^114^ The impact of patterned photoactive layer together with Ag nanograting arrays as the rear electrode in OPVs was attributed to the enhanced light absorption of the photoactive layer through both light diffraction and coupling to SPP modes.^115^ Based on a similar pattern‐transfer process, Hsiao et al. fabricated a simple light trapping scheme featuring 2D periodic granular‐like rear electrode for polymer‐based OPVs.^116^ Peer et al. proposed an advanced light trapping architecture in OPVs with nanostructured metal rear electrode along with an external MLA, which strongly diffracted the incident light on the periodic nanostructure with the excitation of both waveguiding modes and surface plasmon modes.^117^ Accordingly, the photocurrent of such a nano­photonic OPV was enhanced 58% relative to the flat cell, indicating a nearly lossless metal rear electrode.

A comparison of 1D gratings and 2D patterns indicated that the patterned OPVs with 2D nanostructured metal rear electrodes will exhibit superior light harvesting capacity due to a polarization independent plasmonic response.^44^, ^118^, ^119^, ^120^ Such a plasmonic surface could also be achieved through conformal photoactive polymer coating onto a nanoimprinted charge extraction layer.^121^, ^122^ Recently, the moth's eye nanostructured metal rear electrodes consisting of 2D hexagonal periodic grating arrays on top of the photoactive layers were realized by a simple and cost‐effective soft NIL technique, showing a strong potential in enhancing light trapping for OPVs.^64^, ^65^, ^123^, ^124^

In addition to SPPs, localized SPRs excited by randomly nanostructured metal films have been utilized to assist light harvesting in OPVs. To fabricate nonperiodic nanopatterns, Rand et al. introduced PS nanosphere‐templated colloidal lithography for patterning the Ag rear electrode of the nanostructured devices (Figure 8b).^125^ A short‐range ordered non‐closed‐packed array of PS beads was employed by drop casting from an aqueous dispersion on the photoactive layer surface, which was usedas a shadow mask to define nanoholes in a thermally evaporated hole collecting MoO_3_ layer. This pattern was subsequently translated to the Ag layer. After the PS beads were removed by a residue‐free adhesive tape, a nanostructured Ag rear electrode with protrusions that filled in the holes in the MoO_3_ layer was realized, showing the dependency on the diameter of the PS beads and the MoO_3_ thickness. Cheng et al. provided an alternative route to realize the plasmonic Ag rear electrodes with the self‐assembly feature of thermally deposited Ag at the sub‐monolayer thickness, which resulted in the formation of Ag NPs on top of the MoO_3_ layer with the excitation of localized SPRs under the solar illumination.^35^, ^126^ The plasmonic backscattering as well as the Ag NPs‐induced excitation of localized surface plasmons caused an increase in photocurrent of ≈20% without sacrificing electrical properties. Jung et al. also demonstrated highly efficient plasmonic Ag rear electrodes in top‐illuminated OPVs where random hemispherical Ag nanostructured arrays effectively concentrated incident light within the photoactive layer.^127^ This plasmonic Ag array was preferentially fabricated onto glass substrates through the utilization of a simple surface‐tension‐induced agglomeration method. The powerful light trapping via the surface plasmon and scattering effect resulted in a significant increase in PCE from 5.75% to 7.18%. In the solar cell with such plasmonic surface, the key feature lies in the flexibility in tuning the feature size such that the localized SPR can coincide with absorption edges of different photovoltaic absorbers.

An alternative route to achieving SPP or localized SPR enhancement in OPVs is the incorporation of quasi‐random or quasi‐periodic nanostructures into metal electrodes. Unlike the case of perfectly periodic or totally random nanostructures, both broadband absorption enhancement and customizable spectral response can be offered for different photovoltaic semiconductors by quasi‐random nanostructures.^43^, ^128^, ^129^ In this regard, Huang and Sun et al. repurposed Blu‐ray movie discs as quasi‐random nanoimprinting templates for photon management over the solar spectrum (Figure 8c).^130^ The Blu‐ray pattern was successfully imprinted onto the active layer and subsequently translated to the metal electrode of OPVs, leading to enhanced optical trapping. Other than mass‐produced consumables, Chen et al. implemented bio‐inspired quasi‐periodic moth's eye nanostructures to produce a plasmonic surface for light trapping in OPVs.^64^, ^65^ Theoretical analysis verified the role of plasmonic surface of a biomimetic nanostructured metal rear electrode in broadband polarization‐insensitive light trapping. Taking the advantages of regular periodic and random structures, a corrugated metallic grating with multiple superimposed periodical modulations was reported by Dostalek et al. for absorbing light over a broad range of visible wavelengths and angles of incidence.^131^ The multi‐diffractive structure with a three‐diffraction crossed grating corrugation was proved to enhance the absorption in the photoactive film of P3HT:[6,6]‐phenyl‐C61 butyric acid methyl ester (P3HT:PCBM) by 28% over the whole spectra of 400–750 nm and by a factor of 2.9 in the spectral range of 600–750 nm where it was inherently weakly absorbing.

#### 5.2.3 Metasurfaces

**Figure 9:**
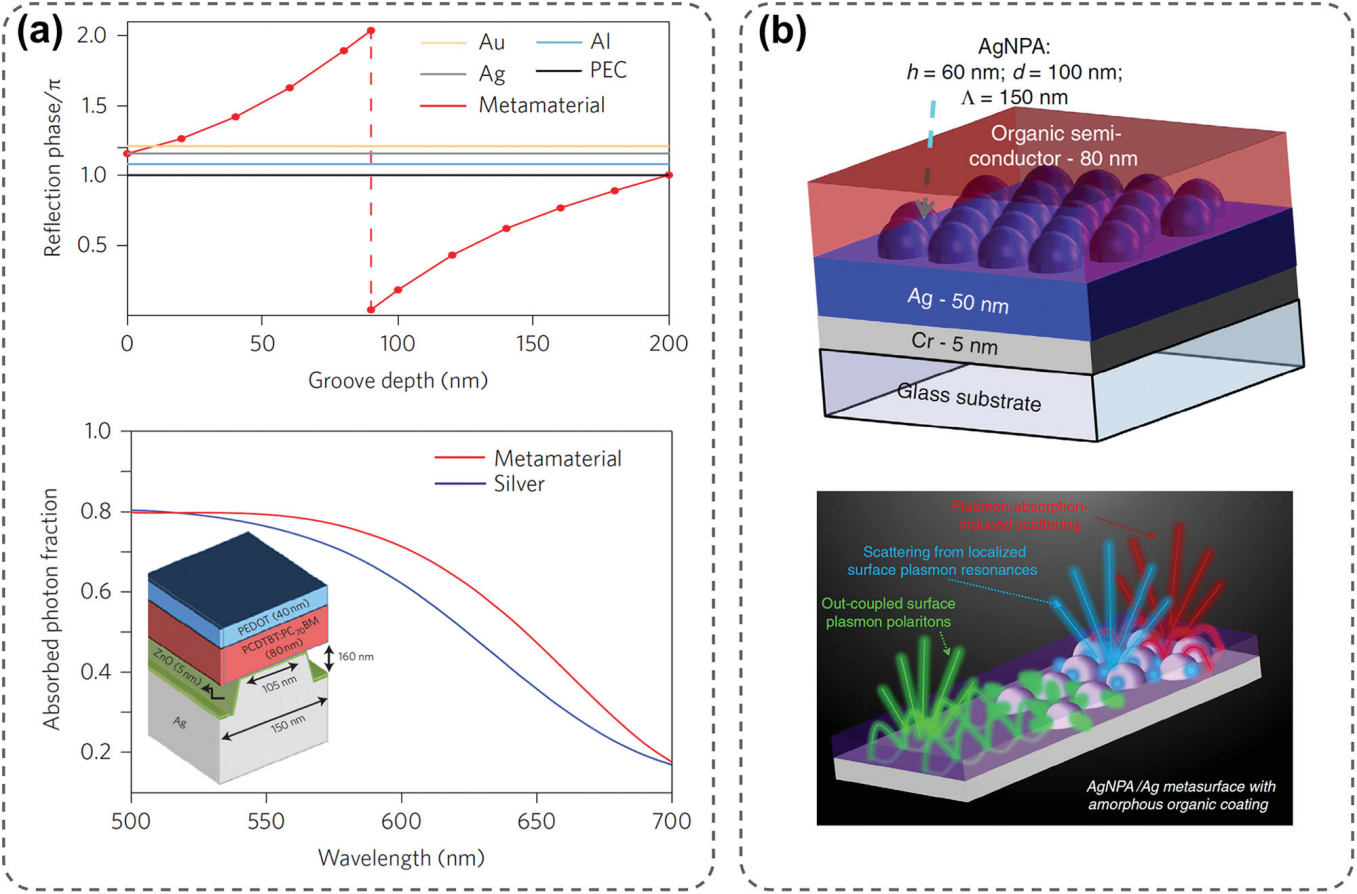
Metamaterial mirrors. a) Reflection phase dependence on groove depth for light 600 nm reflecting from a metamaterial mirror in comparison to perfect electrical conductors (PEC) of Al, Ag and Au, and absorption enhancement of an OPV by implementing a patterned Ag magnetic mirror. Reproduced with permission.^33^ Copyright 2014, Nature Publishing Group. b) A metasurface with silver nanoparticle array and scattering modes supported by the absorber‐coated plasmonic metasurface. Reproduced with permission.^135^ Copyright 2015, Nature Publishing Group.

Very recently, metasurfaces built up from 2D sub‐wavelength metallic building blocks have demonstrated unique responses to the incoming light that transcend those of natural materials.^132^, ^133^, ^134^ For conventional OPVs, a flat metal rear electrode acting as a back mirror to cause phase reversal for reflected light, is highly undesirable since this effect dictates a minimum spacing between the metal rear electrode and the sandwiched photoactive materials and thus posed a fundamental limit to the overall device thickness. However, a metamaterial mirror is favorable to resolve the aforementioned limit by tuning reflection phase from a perfect electric mirror to a perfect magnetic mirror. Esfandyarpour et al. exploited this tunability in reflection phase by optimizing the standing wave profile in planar devices to maximize light‐matter interaction (**Figure** **9**a).^33^ Enhanced light absorption and photocurrent generation by ≈20% over a broad spectral band were achieved in an OPV with a sub‐100 nm thick photoactive layer of the poly[*N*‐9′‐heptadecanyl‐2,7‐carbazole‐alt‐5,5‐(4′,7′‐di‐2‐thienyl‐2′,1′,3′‐benzothiadiazole)]:[6,6]‐phenyl C70‐butyric acid methyl ester (PCDTBT:PC_70_BM) BHJ. Instead of the near field effect commonly used in SPR‐enhanced OPVs, the dominant contribution of such a structure to the absorption enhancement was claimed from the fact that the metamaterial mirror enabled the device to operate closer to resonance by providing the desired reflection phase. As a result, an improved resonant recirculation of the light was achieved, which in turn increased the overall field intensity and the light absorption.^33^

Large‐area metasurfaces composed of Ag NP arrays on Ag thin films coated with various photovoltaic absorber layers were investigated using dark‐field scattering spectroscopy (Figure 9b).^135^ The interactions between organic semiconductors and plasmonic metasurfaces were clarified in three distinct mode types: localized SPRs, propagating SPPs, and plasmon‐absorption‐induced scattering (Figure 9b). By tuning the morphology of the absorber coating and the spectral overlap between the absorber and plasmonic modes, it was possible to control the extinction of the absorption‐induced scattering effect. The SPP mode in this case was trapped for semicrystalline organic absorber coatings and was only scattered to the far field for the amorphous organic absorber‐coated metasurfaces, while the localized SPR and plasmon‐absorption‐induced scattering modes backscatter for all absorber coatings. Further tuning of the coupling between absorber optical transitions and scattering modes was predicted to cause greater absorption enhancements in organic absorber‐coated metasurfaces. The usefulness of the near‐ and sub‐bandgap optical modes could be exploited to a greater extent in completed OPV devices.

### 5.3 Synergistic Effects

#### 5.3.1 Corrugated Electrodes League

**Figure 10:**
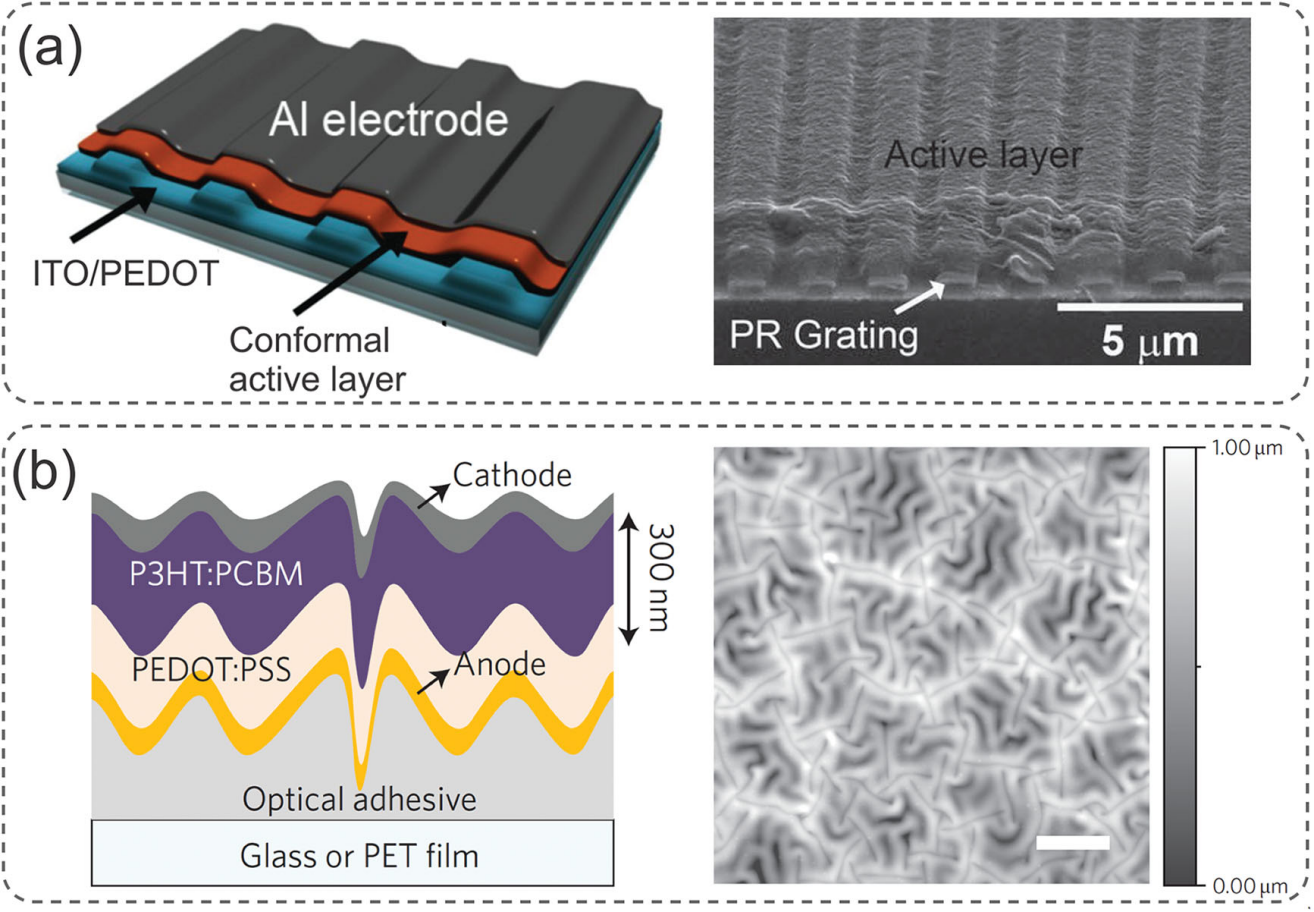
Synergistic corrugation of both contacts. a) OPVs using a textured substrate platform. Reproduced with permission.^136^ b) Device structure using wrinkles and deep folds for photon management. Reproduced with permission.^30^ Copyright 2012, Nature Publishing Group.

Patterning both transparent front electrode and reflective rear electrode with novel optical structures will realize the synergistic effect on light trapping for optimizing the OPV performance. Recently, a series of nanostructured metal grating electrodes for light trapping enhanced OPVs were explored experimentally and theoretically. Nalwa et al. realized high‐efficiency corrugated OPVs based on 1D diffraction ITO gratings, where the grating dimensions with sub‐micrometer height topographies enabled a uniformly thick photoactive layer and a conformally structured metal grating rear electrode (**Figure** **10**a).^136^ Such an OPV possessed broadband light trapping, especially at the band‐edge with an average absorption increase of 100% relative to that of the flat reference cell, yielding the 20% improvement in PCE.

Except for the periodic corrugation of the electrode pairs in OPV structures, another effective route is the use of self‐organizing wrinkles and deep folds as photonic structures that were formed on polymer surfaces induced by mechanical stress (Figure 10b).^30^ The OPVs constructed on such surfaces can effectively guide and retain light within the photoactive regions, resulting in substantial improvement in light trapping efficiency. Intriguingly, a vast increase in EQE more than 600% was obtained in the near‐infrared region for the photoactive materials with minimal photon absorption. The photoactive range of solar energy conversion was thus extended by more than 200 nm with the presence of wrinkles and folds.

In addition, surface plasmon‐mediated absorption enhancement was demonstrated in bottom‐up corrugated small‐molecule OPVs with 1D, 2D periodic gratings, or dual‐periodic grating.^137^, ^138^ A particular utilization of periodically corrugated electrodes is to match photocurrents of sub‐cells in tandem OPVs through coupling between SPPs and microcavity modes.^49^ The anti‐crossing behavior between the SPP and microcavity modes within the device was identified in enhancing the absorption of the back sub‐cell, and thereby achieving a balanced photocurrent of front and back sub‐cells. The periodical corrugation of dual metal electrodes resulted in 10.4% enhancement in the photocurrent, and 11.3% enhancement in the PCE in the double‐junction small molecule OPVs.

#### 5.3.2 Optical Microcavity Resonance

**Figure 11:**
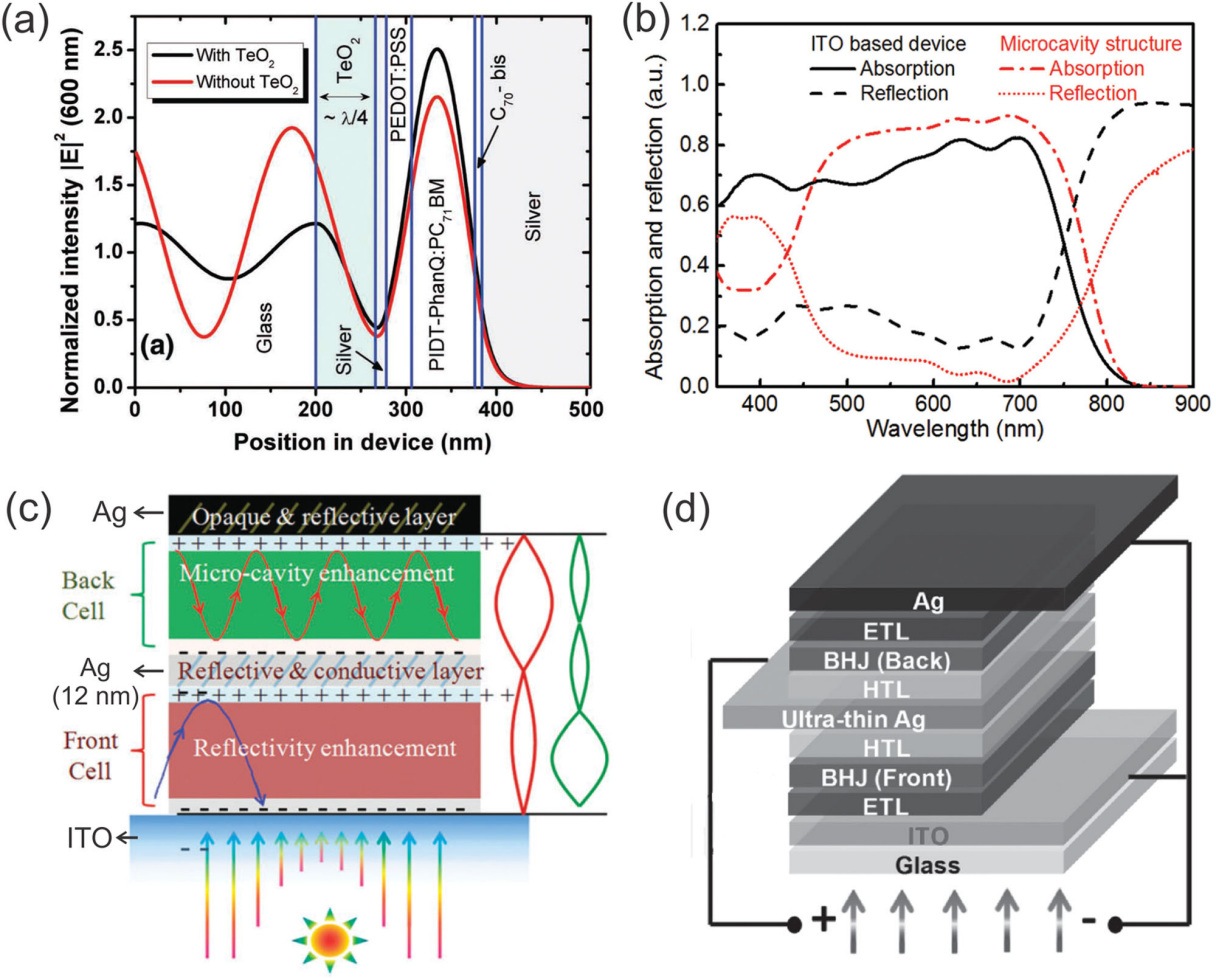
Optical microcavity resonance. a) A dielectric film as light modulation layer for tuning the electric field intensity. Reproduced with permission.^148^ b) Optical properties of OPVs in microcavity configuration. Reproduced with permission.^149^ c) Modification of optical field distribution in series‐connected tandem OPVs with a microcavity formed in the back sub‐cell and reflection of high‐energy photons in front sub‐cell. Reproduced with permission.^150^ Copyright 2015, Royal Society of Chemistry. d) Parallel tandem OPVs with microcavity‐enhanced light trapping. Reproduced with permission.^151^

In recent years, the integration of a microcavity structure into OPVs has been demonstrated effectively to improve light trapping inside the device.^139^, ^140^, ^141^, ^142^, ^143^ A microcavity‐enhanced OPV usually consists of two planar electrodes with appropriate reflectivity to produce microcavity resonance. To develop efficient microcavity‐enhanced OPVs, a homogeneous metal thin film is required to be fabricated simultaneously with the reduction of optical reflection loss. In this regard, some dielectric layers such as high‐refractive‐index materials (e.g., MoO_3_, TeO_2_, WO_3_) were utilized to manipulate the local electromagnetic field distribution in the microcavity cell.^144^, ^145^, ^146^, ^147^ By introducing a highly transparent TeO_2_ film in resonance with the Ag‐Ag microcavity, the total reflection of an ultrathin Ag film was theoretically reduced and an increased electric field was thus observed within the active layer (**Figure** **11**a).^148^ Based on this design, Huang et al. fabricated top‐illuminated OPVs with microcavity structure in comparison with ITO‐based cells (Figure 11b).^149^ The absorption of the photoactive layer in microcavity OPV was stronger than that of the ITO‐based device in the 450–800 nm wavelength region, while slightly decreased absorption was observed in the region below 450 nm due to the noncoherent interference. Consequently, the fine‐tuned microcavity configuration enhanced light trapping of ITO‐free OPV devices, leading to 11% improvement in *J*_sc_ and 10% enhancement in PCE to 10.5% compared to the typical ITO‐based devices.

By virtue of microcavity configuration, Zuo et al. developed an efficient interconnecting layer based on an ultrathin reflective Ag film for series‐connected microcavity tandem OPV (Figure 11c).^150^ Such a tandem OPV benefited from the optimized distribution of optical field intensity through strong reflectivity generated by the ultrathin Ag layer in the front sub‐cell and the formation of a microcavity in the back sub‐cell, yielding a high PCE of ≈11% and high summed EQE over 90%. The same research group also demonstrated high performance parallel tandem OPVs comprising a semi‐transparent front cell and a microcavity assisted top‐illuminated back cell (Figure 11d).^151^ Microcavity effects induced by an ultrathin intermediate Ag transparent electrode facilitated the light trapping, resulting in a record PCE of 9.2% for parallel tandem OPVs.

## 6 Nanostructuring of Charge Extraction and Photoactive Layers

### 6.1 Metal NPs‐Mediated Plasmonic Enhancement

#### 6.1.1 Modification of Charge Extraction Layers

Charge extraction layers (CELs) typically having a few tens of nanometers play an important role in OPVs, which can favor the efficient extraction of generated carriers from organic photoactive layers into the electrodes.^152^, ^153^ Regardless of the charge extraction capability, engineering the CELs by tuning their thickness and geometric structures can favor the light trapping inside the cells. For example, the CELs can function as an optical spacer to alter the spatial distribution of the optical electric field inside the cells without optical absorption loss, thereby putting the photoactive layer in a more favorable region of the optical electric field.^154^

**Figure 12:**
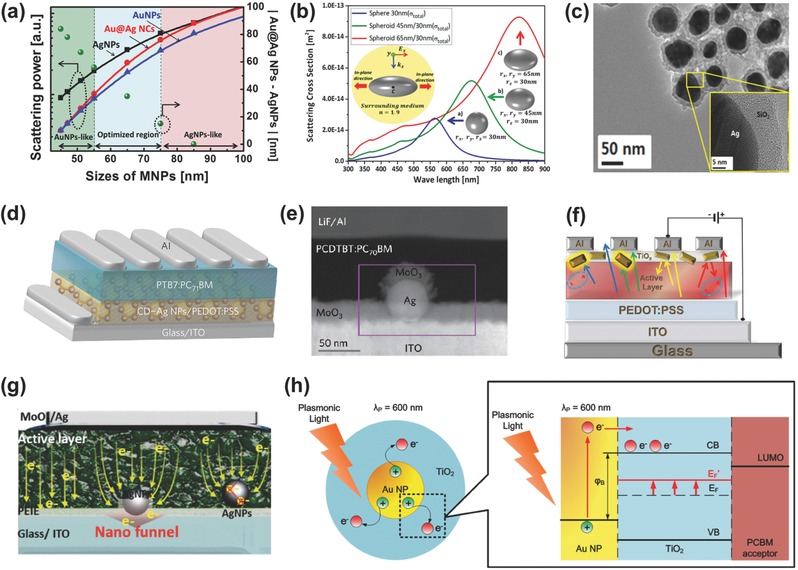
Plasmonic enhancement with metal NPs embedded in charge extraction layers. a) Scattering dependence on particle sizes and materials. Reproduced with permission.^158^ Copyright 2014, American Chemical Society. b) Influence on the shape of metal NPs. Reproduced with permission.^163^ Copyright 2014, American Chemical Society. c) Effect of the capping layer on scattering efficiency. Reproduced with permission.^169^ Copyright 2013, American Chemical Society. d) Hole extraction layer embedded with carbon‐dot‐supported Ag NPs. Reproduced with permission.^31^ Copyright 2013, Nature Publishing Group. e) Hole extraction layer embedded with aerosol‐derived Ag NPs. Reproduced with permission.^173^ Copyright 2014, American Chemical Society. f) Electron extraction layer embedded with Au nanorods. Reproduced with permission.^175^ g) The enhanced charge transport and extraction assisted by the metal NP/dielectric hybrid layer. Reproduced with permission.^183^ h) Plasmon‐electrical effect on charge injection process. Reproduced with permission.^184^

To realize a plasmonic‐mediated layer for the light harvesting enhancement, the incorporation of metal NPs with different materials, sizes, shapes, concentrations or distributions into charge extraction layers (CELs) has recently been extensively investigated.^155^, ^156^, ^157^ Baek et al. compared the effect of Au NPs, Ag NPs, and Au@Ag core‐shell nanocubes on the optical scattering properties (**Figure** **12**a).^158^ It was found that Au@Ag nanocubes (NCs) could be a highly efficient hybrid plasmonic material to achieve both a high scattering efficiency of Ag NPs and a feasible broadband absorption enhancement of Au NPs. By optimizing the core size of Au NPs and the Ag shell thickness of the designed hybrid naocubes, it could minimize the blue shift of the localized SPR while maximizing the scattering power of metal NPs. The OPVs with Au@Ag nanocubes embedded in a PEDOT:PSS hole extraction layer (HEL) showed 2.2‐fold absorption enhancement at wavelengths of 450–700 nm compared to Au NPs due to the amplified plasmonic effect.^158^ Cooperative plasmonic effect of Au and Ag nanostructures on enhancing light harvesting was also obtained by directly mixing Au and Ag NPs in PEDOT:PSS or depositing Au–Ag alloy NPs onto ITO anode.^159^, ^160^ For example, Lee et al. systematically analyzed the size dependent plasmonic forward scattering effect by incorporated size‐controlled Ag NPs (diameter: 10–100 nm) into the PEDOT:PSS HEL of OPVs, and the origin was then interpreted by visualizing the scattering field with near field optical microscopy.^161^ Fleetham et al. directly deposited Ag NPs of various nominal thicknesses on ITO, and performed the post‐annealing treatment to increase their size in radius, which was followed by employing the PEDOT:PSS HEL on Ag NPs‐coated ITO substrates to alter the dewetting behavior of Ag NPs.^162^

The impact of NP shapes on the directionality of scattering and localized SPRs of metallic spheroidal nanostructures was investigated by Park et al.^163^ Figure 12b shows the wavelength dependence of total scattering cross‐section of an isolated Ag spheroidal NP immersed in a uniform polymer medium when illuminated by a plane wave propagating in the direction of the spheroid minor axis, at several different eccentricities: sphere (*e* = 0), slight oblate spheroid (*e* = 0.75), and optimized spheroid (*e* = 0.89). It is found that increasing the spheroid eccentricity can provide an overall increase of scattering power across the entire spectral range with a broadened, red‐shifted localized SPR.^163^ Sum et al. demonstrated the efficiency enhancement in plasmonic OPVs with the integration of large‐area periodic Ag nanotriangle arrays that were fabricated using the cost‐effective, high‐throughput nanosphere lithography technique.^164^ The improvements of the PCE from 4.24 to 4.52% with ≈12% enhanced *J*_sc_ were attributed to an increase in exciton generation induced by the strong local electric field and the scattering generated by localized SPRs of the hexagonal nanotriangle arrays. Similarly, self‐assembled Au nanopyramid arrays fabricated through the PS spheres template were demonstrated by Ren et al. to greatly enhance the photocurrent of OPVs due to the plasmonic near field effect, yielding an increase of up to 200% in PCE.^165^ Oo et al. also exploited solution‐processed ultrafine Au nanowires as plasmonic antennae in OPVs.^166^ An increased *J*_sc_ by 23.2% was then obtained by optimizing the spacer layer thickness for extending the evanescent field into the photo­active layer and varying the geometry of the Au nanowires bands for favoring the enhanced scattering. The geometry effects of Au NPs with different morphologies (i.e., star, rod, sphere) in the PEDOT:PSS HEL were compared, showing 29% increase in PCE for Au nanostars, 14% for Au nanorods, and 11% for Au nanospheres, respectively.^167^ The difference in the efficiency enhancement was ascribed to the strongest localized SPR effect of Au nanostars with large size and sharp features. In addition, Au NPs of various sizes and shapes, featuring different localized SPR wavelengths were blended into the PEDOT:PSS layer of OPVs, leading to efficient enhancement of light absorption by ≈17% due to the localized SPR‐induced near field enhancement and less light scattering effect.^168^

**Figure 13:**
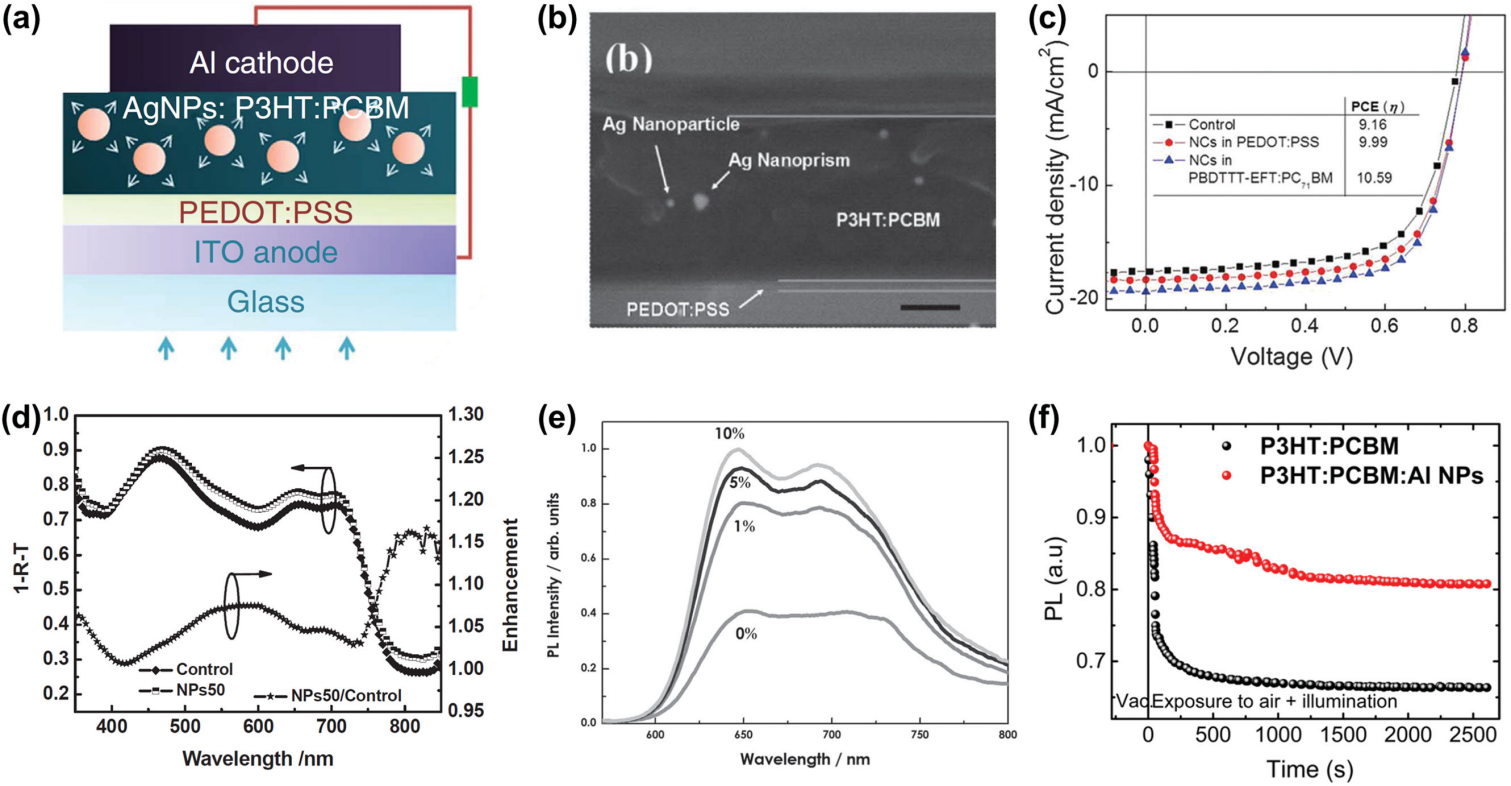
Plasmonic enhancement with metal NPs embedded in photoactive layers. a) A typical plamsonic OPV with Ag NPs in the BHJ photoactive layer. Reproduced with permission.^186^ Copyright 2013, Nature Publishing Group. b) Incorporation of mixed Ag NPs and nanoprisms in the active layer. Reproduced with permission.^191^ c) Photovoltaic performance with embedded Au@Ag@SiO_2_ nanocuboids. Reproduced with permission.^200^ Copyright 2016, Royal Society of Chemistry. d) Influence of optical properties with embedded Au NPs. Reproduced with permission.^204^ e) Photoluminescence (PL) properties with Au–Ag alloy NPs. Reproduced with permission.^210^ f) Transient PL response with Al NPs. Reproduced with permission.^213^ Copyright 2015, American Chemical Society.

In spite of metal NPs‐mediated light absorption enhancement, the incorporation of bare metal NPs has the potential to cause electrical losses due to charge trapping and exciton quenching near the vicinity of the metal surface. The strategies of coating a thin dielectric layer onto metal NPs or supporting metal NPs on fixed templates have been demonstrated to benefit the plasmonic absorption enhancement. For example, the silica shell in Ag@SiO_2_ could preserve the localized SPR effect of Ag NPs by preventing oxidation of the Ag core under ambient conditions (**Figure** **13**c).^169^ Similarly, large size Ag@SiO_2_ NPs were incorporated within the HEL, partly protruding into the active layer.^170^ This solution not only increased light absorption in the photoactive layer by simultaneously inducing the effect of far field scattering and laterally distributed localized SPR, but also promoted hole extraction by reducing hole trapping and exciton quenching in the bare Ag surface.

Apart from dielectric shells, Kim et al. designed carbon dot‐supported Ag (CD‐Ag) NPs using the carbon dots both as a reducing agent and a template to realize versatile localized SPR (Figure 13d).^31^ The clustering effect of Ag NPs on the carbon dots resulted in broadband absorption from electric field enhancement without any changes in the size or shape of the NPs. In addition, the fast reduction process of metal salts to form metal NPs on the surfaces of carbon dots was guaranteed by the excellent electron‐donating capability of photoexcited carbon dots. The carbon dots‐induced templating and clustering effects dramatically strengthened the localized SPR, allowing significant light absorption with enhanced PCE up to 8.31% and an internal quantum efficiency (IQE) of 99% in PTB7‐based OPV.^31^ Graphene and related 2D nanomaterials have attracted extensive research interest in the photovoltaic applications due to their unprecedented material properties.^171^ Recently, graphene oxide (GO) has been utilized as a template for metallic nanostructures (e.g., Au NPs) with relatively controlled dispersion and density to trigger a plasmonic effect in OPVs.^45^ Based on the incorporation of Au NPs‐coated GO nanocomposites into the PEDOT:PSS HEL, the light absorption and exciton generation rate of the photoactive layer was enhanced with the excitation of the localized SPR of Au‐GOs along the photoactive layer/PEDOT:PSS interface. Alternatively, Chen and Ye et al. directly utilized a hybrid structure of Au NPs decorated on 2D molybdenum disulfide nanosheets (MoS_2_@Au) as multifunctional HEL to facilitate hole extraction at the anode and light trapping enhancement with coupled plasmonic near field.^172^

Different from the mixture of metal NPs and PEDOT:PSS, Jung and co‐workers constructed a nanobump assembly of MoO_3_‐covered Ag NPs adjacent to the photoactive layer, where the NPs with precisely controlled concentration and size were generated by atmospheric evaporation and mobility classification methods and the MoO_3_ layer was used to enclose Ag NPs via vacuum thermal evaporation to isolate the undulated active layer (Figure 12e).^173^ The OPV constructed on this composite layer yielded a PCE enhancement of 18% due to the enhanced light scattering and multi‐reflection effects arising from the nanobump structure combined with the undulated photoactive layer in the visible and near‐infrared regions. The effect of localized SPR was also realized by incorporating Au and/or Ag NPs into the TiO_2_ electron extraction layer (EEL), leading to 20.7% enhancement in *J*_sc_ and a maximum PCE of 7.52% with an optimized concentration of Ag NPs.^174^

Complementary to forward scattering by localized SPRs, plasmonic backscattering effects are also verified to effectively increase optical absorption in OPVs. As Kymakis et al. demonstrated,^175^ efficient light trapping by incorporating Au nanorods in the TiO_2_ EEL of OPVs was achieved with a PCE improvement ratio of 18% (Figure 12f). A dual‐plasmonic effect was claimed to be responsible for the broad and uniform increase in EQE spectra: the far field scattering of Au nanorods inside the TiO_2_ layer and the near field SPR of the nanorods that penetrated into the photoactive layer. Similarly, a diameter‐controlled thermally evaporation method was used to realize the doping of Au NPs into the WO_3_ HEL in inverted OPVs, in which the difference in surface energies between Au and WO_3_ resulted in the Au growing up from nucleation, isolated island, aggregation of metal islands to continuous films in the process of evaporation.^176^ The corresponding OPVs with an optimized 8 nm‐thick Au shown a dramatically increased PCE from ≈4.67% to ≈6.63% due to the plasmonic backscattering effect and modified electrical characteristics. Taking into account the limited spectral range response with single type of metal NPs, dual metal NPs (i.e., Au and Ag NPs) were presented near the rear electrode of OPVs for SPR‐induced absorption enhancement in almost entire absorption range of the photoactive layer by both Ag NPs (350−450 nm) and Au NPs (450−600 nm), leading to a significant improvement of ≈43% in PCE.^177^

Recently, high‐efficiency OPVs were achieved via the incorporation of plasmonic metal NPs into both HEL and EEL to enhance light trapping. By engineering both the rear and front interfacial layers of the OPV with different sizes of Au NPs, Jen and Chen et al. demonstrated that the PCE of the devices was improved from 6.65% to 7.50%.^178^ In contrast to this modest enhancement, Jen and his colleagues utilized tunable Ag nanoprisms with optical properties superior to those of Ag and Au nanospheres, allowing for an enhancement of 18% in PCE from 7.7% to 9.0%.^179^ Most recently, Jen et al. further integrated the plasmonic effect into the microcavity architecture of ITO‐free flexible OPVs, and demonstrated a significant enhancement in PCE from 8.5% (reference microcavity device) to 9.4% by carefully controlling the sizes of embedded Ag nanoprisms in dual CELs.^180^ Such an improvement resulted from the spectral matching between localized SPR peaks of Ag nanoprisms and relatively low absorption response of the photoactive layer induced by the microcavity effect. Another route to achieve the effect of dual plasmonic resonances was provided by thermally depositing Au nanodots between the photoactive layer and cathode, and incorporating octahedral Au NPs within the PEDOT:PSS HEL.^181^ It was found that Au NPs‐induced local field enhancement was favorable for both light absorption and photo‐induced charge separation processes, while Au nanodots enhanced the parasitic absorption of light and an elevated degree of exciton dissociation. Yang et al. demonstrated the plasmonic effects in an inverted tandem OPV configuration by blending Au NPs into a solution processed interconnector layer, which can simultaneously enhance the optical absorption of both top and bottom sub‐cells with a 20% improvement of overall PCE via plasmonic near‐field enhanced light concentration of Au NPs.^182^ This plasmonic enhanced interconnector suggested a potential of achieving highly efficient multi‐junction OPVs by integrating the plasmonic effect with conventional device structure.

Besides the well‐recognized optical gain of embedded metal NPs in OPVs, plasmonic metal nanomaterials could be utilized to improve the electrical properties by assisting the charge transport and extraction processes of OPVs.^183^, ^184^, ^185^ For example, a metal/dielectric hybrid layer with the combination of Ag NPs and a polyethylenimine ethoxylated (PEIE) dielectric layer could provide a short path and funneled charge carriers to the cathode, thus effectively increasing the electron extraction (Figure 12g).^183^ As a result, this hybrid layer platform could maximize the cell IQE to nearly 100% with a resulting PCE of 10.1% and doubled half‐efficiency lifetime. As shown in Figure 12h, the integrated optical and electrical model was constructed by taking into account the hot carrier tunneling probability and extraction barrier between TiO_2_ and the active layer.^184^ The strong charge injection of plasmonic excited electrons from NPs into TiO_2_ contributed to the enhanced charge extraction under plasmonic illumination. Such a mechanism can be used to lower the effective energy barrier and facilitate charge transport in OPVs by trap filling in TiO_2_.^185^

#### 6.1.2 Modification of Photoactive Layers

Considering the evanescent characteristics of localized SPRs, blending metal NPs into photoactive layers is preferred in many plasmonic‐enhanced OPVs (Figure 13a).^186^ Early in 2005, the introduction of metal NPs (e.g., Au or Ag NPs) into the organic absorber matrix was reported as an approach to modifying the nanophase of BHJs from the perspective of improved electrical conductivity via the introduction of “dopant” levels.^187^ In 2009, enhanced light absorption up to 50% was experimentally obtained for a photoactive layer using poly(2‐methoxy‐5‐(20‐ethyl‐hexyloxy)‐1,4‐phenylenevinylene):(6,6)‐phenyl‐C61‐butyric‐acid‐methyl ester (MEHPPV:PCBM) BHJ containing Ag NPs, which was recognized and interpreted theoretically by SPRs.^188^ The following year witnessed up to 3 times enhanced charge generation and long‐lived photogenerated charge carriers in optically thin P3HT:PCBM films by plasmon‐resonant Ag nanoprisms (≈40‐100 nm edge length).^189^ A 3D modeling design of a hexagonal periodic Ag nanosphere array in photoactive layers was later introduced, showing broadband optical absorption enhancement with a weak polarization dependence on incident light owing to the light concentration effect by localized SPRs.^190^

To resolve the limit of narrow resonant absorption, a combination of Ag nanomaterials of different shapes was proposed by Choy et al. for broadband plasmonic enhancement in OPVs, and simultaneous excitation of many plasmonic low‐ and high‐order resonances modes was experimentally and theoretically demonstrated to be material‐, shape‐, size‐, and polarization‐dependent (Figure 13b).^191^ To further understandings of the plasmonic scattering effect, various metal nanostructures in different shapes integrated into photoactive layers were also investigated, including Ag nanowires,^192^ Au nanorods,^193^ Au arrow‐head nanorods,^194^ and Au particles in the styles of nanoscale cubes, rhombic dodecahedra, edge‐ and corner‐truncated octahedra, and triangular plates.^195^

However, the effect of metal NPs in the photoactive layers on the performance enhancement remains a debate. As demonstrated by Heeger and co‐workers, the OPV devices with Ag NPs‐aggregated clusters embedded into photoactive layers exhibited enhanced light trapping by the scattering and excitation of localized SPRs,^196^ while the incorporation of Au NPs with a truncated octahedral structure into the photoactive layers resulted in enhanced light absorption due to Au NPs‐induced light scattering rather than plasmon‐induced light concentration at specific wavelengths.^197^ Other challenge for blending metal NPs into the photoactive layers involves the induced performance degradation with the metal NPs as an additive to the active layer,^198^ which is accompanied with reduced carrier density and increased recombination from carriers trapped on the embedded Ag NPs.^199^ In this regard, Wu et al. provided evidence of traps responsible for such degradation in plasmonic OPVs through comprehensive transient optical spectroscopy and electrical characterization on the device with oleylamine‐capped Ag NPs blended in the P3HT:PCBM active layer.^186^ The increased trap‐assisted recombination of photogenerated excitons was confirmed after an initial increase promoted by the presence of Ag NPs.

To circumvent the challenges involved in photoactive layers, coating metal NPs with insulating shells has been proposed for optical absorption enhancement in OPVs. The insulator shell layer added onto metal core NPs is commonly considered to provide an electrically insulating surface that does not interfere with carrier generation and transport inside the active layer, and also to eliminate the concern about exciton quenching by avoiding direct contact between metal cores and the photoactive layer. Taking into account trap‐assisted exciton recombination, Yan et al. incorporated the Au@Ag@SiO_2_ core–shell nanocuboids into photoactive layers, showing that multimode localized SPRs could be tuned to match the light absorption spectra of the OPVs by changing the particle size and the Ag shell thickness (Figure 13c).^200^ The device performance was substantially improved to a high PCE of 10.59% due to both light scattering and near field enhancement induced by the nanocuboids. Choi et al. also demonstrated that OPVs incorporating SiO_2_‐coated Ag NPs in the polymer‐based photoactive layer achieved an enhancement of ≈19% in PCE via additional light absorption and scattering effects over a broad spectral range of 400−700 nm.^169^ Similarly, an increase in light harvesting efficiency was realized by incorporating octadecyltrimethoxysilane (OTMS)‐functionalized, spectrally tuned, Au@SiO_2_ core‐shell nanospheres and nanorods into the active layers of OPVs with various absorbers.^201^ Functionalization of the Au@SiO_2_ core‐shell NPs with the OTMS organic ligand benefits the transfer of the composite NPs from an ethanol solution into an OPV polymer‐compatible solvent, such as dichlorobenzene. By virtue of such encapsulated metallic nanostructures, Chen et al. introduced large size Au@SiO_2_ core‐shell NPs composed of Au NPs with 70 nm diameter coated by a ≈50 nm thick SiO_2_ shell into polymer‐based OPVs, resulting in a ≈16% efficiency enhancement by localized SPR effect without sacrificing electrical characteristics.^202^ In addition to insulated shells, Liu et al. proposed a hybrid plasmonic nanostructure using Ag NPs‐decorated 1D TiO_2_ nanorods to enhance the photocurrent of OPVs through a strong localized electric field and an enhanced charge transport channel.^203^

The synergistic effect of plasmonic enhancement could be achieved by modifying different functional components in an OPV device. Choy and Hou et al. fabricated the OPV with dual metallic nanostructures consisting of Au NPs embedded in the active layer and Ag nanograting (period ≈750 nm) as the plasmonic mirror enabled positive electrical effects and broadband absorption enhancement by the collective excitation and hybridization of localized SPR, SPP, and Floquet modes (Figure 13d).^204^ Enhanced light absorption of OPVs with dual plasmonic nanostructures was determined mainly around the infrared region from the directly measured transmission and reflection characteristics. As a result, the accumulated optical and electrical improvements by dual metallic nanostructures led to a considerable efficiency enhancement of ≈15.8%, achieving a PCE of 8.79% in inverted OPVs. Li et al. proposed an alternative dual plasmonic scheme to achieve the broadband enhancement of OPVs, including the selection of Ag grating with 600 nm period as the reflective rear electrode and the incorporation of Au NPs into the EEL instead of active layer.^205^ Collectively plasmonic effects of SPPs and plasmon‐enhanced forward scattering were obtained in these OPVs, achieving absorption enhancement in a wider wavelength range of 350–800 nm compared to that of only using Ag back grating or Au NPs in photoactive layers. As a consequence, an appreciable light harvesting enhancement in photoactive layers enabled a significant efficiency improvement of ≈22%, yielding a maximum PCE of 9.62% and an average PCE of 9.34% as compared to 7.7% for control cells without any plasmonic nanostructures.

Instead of the utilization of both localized SPR and SPP modes, dual localized SPR enhancements totally by metal NPs have been demonstrated in enhancing light harvesting for OPVs. For example, simultaneously incorporating Au nanospheres into the HEL and Au@SiO_2_ nanorods into the photoactive layer led to superior broadband absorption improvement in a solution‐processed small molecule OPV with 31% enhancement in PCE up to 8.72%.^206^ Concurrent enhancements of charge generation, dissociation, and transport properties of OPVs were demonstrated by incorporating Au NPs into the PEDOT:PSS HEL and Au NPs‐decorated nitrogen‐ or boron‐doped carbon nanotubes (CNTs) into the PTB7:PCBM photoactive layer.^207^ Accordingly, dual‐positional Au NPs enabled localized SPR‐induced charge generation and dissociation, while the heteroatom‐doped CNTs promoted charge selective transport and better local ordering of photoactive polymers. In addition, the efficiency improvement in OPVs was demonstrated by incorporating Au NPs into all polymer layers, where Au NPs in PEDOT:PSS mainly contributed to better hole extraction, and Au NPs in the photoactive layer benefited optical absorption and balanced charge transport by plasmon resonances with strong near field distributions.^208^, ^209^

Compared to single element metal NPs, metallic alloy nanostructures equally exhibited the great plasmonic enhancement in light trapping. Chen et al. reported an one‐pot synthesis of large size and high quality Au:Ag alloy NPs with well controlled compositions, and the utilization of 1% Au_11_Ag_89_ alloy NPs embedded in the photoactive layer of OPVs resulted in 31% PCE improvement due to the enhancement of both light‐trapping and charge transport in the active layer (Figure 13e).^210^ Considerable enhancement of the photoluminescence (PL) emission upon increasing the concentrations of nanoalloys suggested the reduction of exciton quenching on the NPs' surface due to the presence of the capping insulating surfactants and the excitation of localized SPRs that enhanced the light excitation rate and thereby optical absorption of polymer semiconductors.

Recent studies suggested that aluminum (Al) NPs held the potential to yield significantly greater plasmonic enhancement than Ag or Au, since much higher plasma frequency of Al ensured a better overlap between plasmonic resonance and absorption band of organic semiconductors.^211^, ^212^, ^213^ Stratakis and Kymakis et al. showed that the addition of highly stable Al NPs into the photoactive layer could simultaneously enhance efficiency and stability of OPVs in different absorber systems.^213^ As shown in Figure 13f, the retarded PL intensity decay rate by the Al NPs dispersed in the polymer blend indicated a high possibility that the local energy environment might attract long‐lived excitons toward Al NPs where the triplet exciton–Al NP interaction can easily take place, as previously shown in Figure 2b.

### 6.2 Photonic Structures‐Integrated Photoactive Layers

In spite of the incorporation of metal NPs, dielectric nanostructures provide an alternative route to enhance the light scattering capacity for efficient OPVs.^214^, ^215^, ^216^, ^217^ For example, the integration of monodispersed PS nanospheres into poly(3,4‐ethylenedioxythiophene):poly(styrenesulfonate) (PEDOT:PSS) films can induce strong forward scattering effect, or create a randomly nano‐textured metal rear electrode.^214^, ^215^, ^216^ The incorporation of SiO_2_ NPs into photoactive layer was also reported to result in 13% increase in PCE, while PCE was increased by 20% when SiO_2_ NPs were incorporated into charge extracting layers.^217^

**Figure 14:**
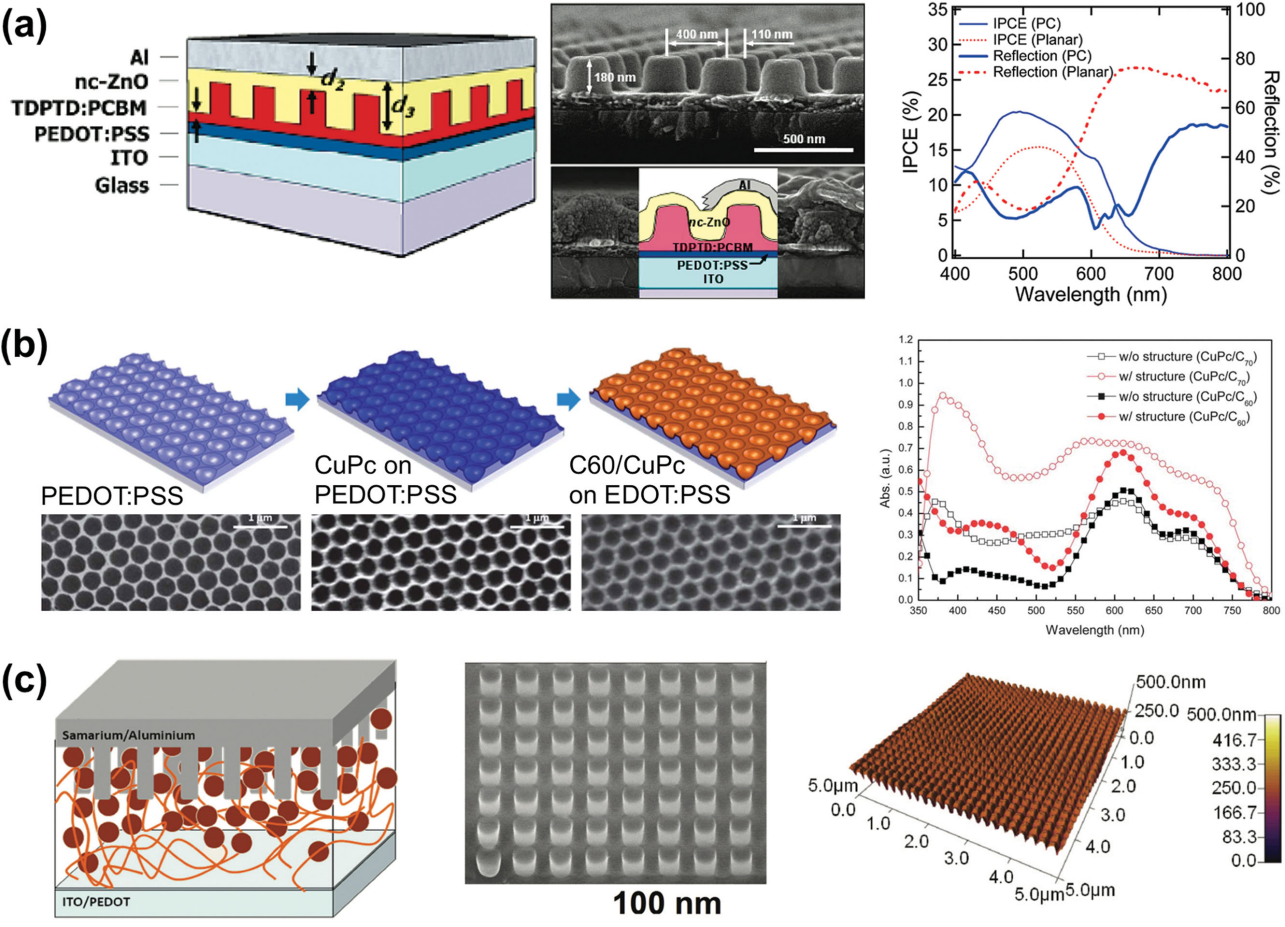
Implementation of photonic structures in photoactive layers. a) The OPV geometry with hexagonal array of BHJ columns and the influence on photovoltaic and reflection properties. Reproduced with permission.^219^ Copyright 2009, American Chemical Society. b) Nanostructured photoactive layer on PEDOT:PSS nanobowl array, and the modification of absorption characteristics. Reproduced with permission.^222^ Copyright 2013, Royal Society of Chemistry. c) Nanostructured OPV, silicon moulds and the structured photoactive layer. Reproduced with permission.^223^

For the application of large‐area OPVs, the implementation of photonic structures directly patterned on the CELs and photoactive layers through continuous and scalable fabrication techniques are highly desirable. Photonic structure designs in photoactive layers of OPVs have attracted considerable attention, and theoretical considerations of photonic geometries suggested light harvesting efficiency enhancement with a periodic nanostructured polymer absorber film due to the strongly enhanced light‐matter interaction with maximized density of Bloch modes.^218^ The most commonly used echniques for structuring the photoactive layer in OPVs include hot‐embossing, surface relief grating formation by laser interference lithography, and NIL. For example, Ko et al. reported an OPV with a highly ordered photonic crystal (PC) nanostructure embossed in the photoactive layer using a materials‐agnostic process, enabling a 3‐fold enhancement of the absorption in specific regions of the solar spectrum in part through multiple excitation resonances (**Figure** **14**a).^219^ As shown in Figure 14a, this PC geometry with a highly ordered array of nanoscale columns stimulated light absorption in the entire absorption wavelength range and hence the PCE increase of ≈70%. In addition, the comparison of light trapping effect between 1D and 2D periodic PC nanostructures indicated that absorption enhancement originated from band edge excitation of quasi‐guided modes, but the enhancement ratio varied with the control of physical dimensions.^220^ A theoretically optical mapping of the distribution of electric field intensities in periodic PC‐nanostructured photoactive layer exhibied the remarkably modified and concentrated light for the gradient type PC hollow, implying strongly enhanced absorption in the PC‐structured photoactive layer due to better optical impedance matching and prolonged optical path.^64^, ^221^

Wei et al. constructed bilayer OPV by using electrochemical deposition (with PS beads as the template) to fabricate a PEDOT:PSS HEL with nanobowl array structures on ITO substrate (Figure 14b).^222^ By implementing this spatial structure, a high absorption of incident light in small‐molecule‐based OPVs was obtained with respect to the simultaneously increased light path in the active layer and exciton dissociation efficiency by enlarging the donor–acceptor interface. The photocurrent generated by such a bilayer OPV was increased by ≈90% in comparison to an equally thick planar control active layer. Pandey et al. reported a facile approach to structure the polymer BHJ in OPVs, enabling higher efficiencies with increased photocurrent (Figure 14c).^223^ Soft contact imprinting was employed to structure the top surface of the photoactive layer by using different Si molds. The substantial and reproducible photocurrent enhancements were derived from both enhanced optical trapping and improved electron extraction at the structured metal/organic photoactive layer interface.

**Figure 15:**
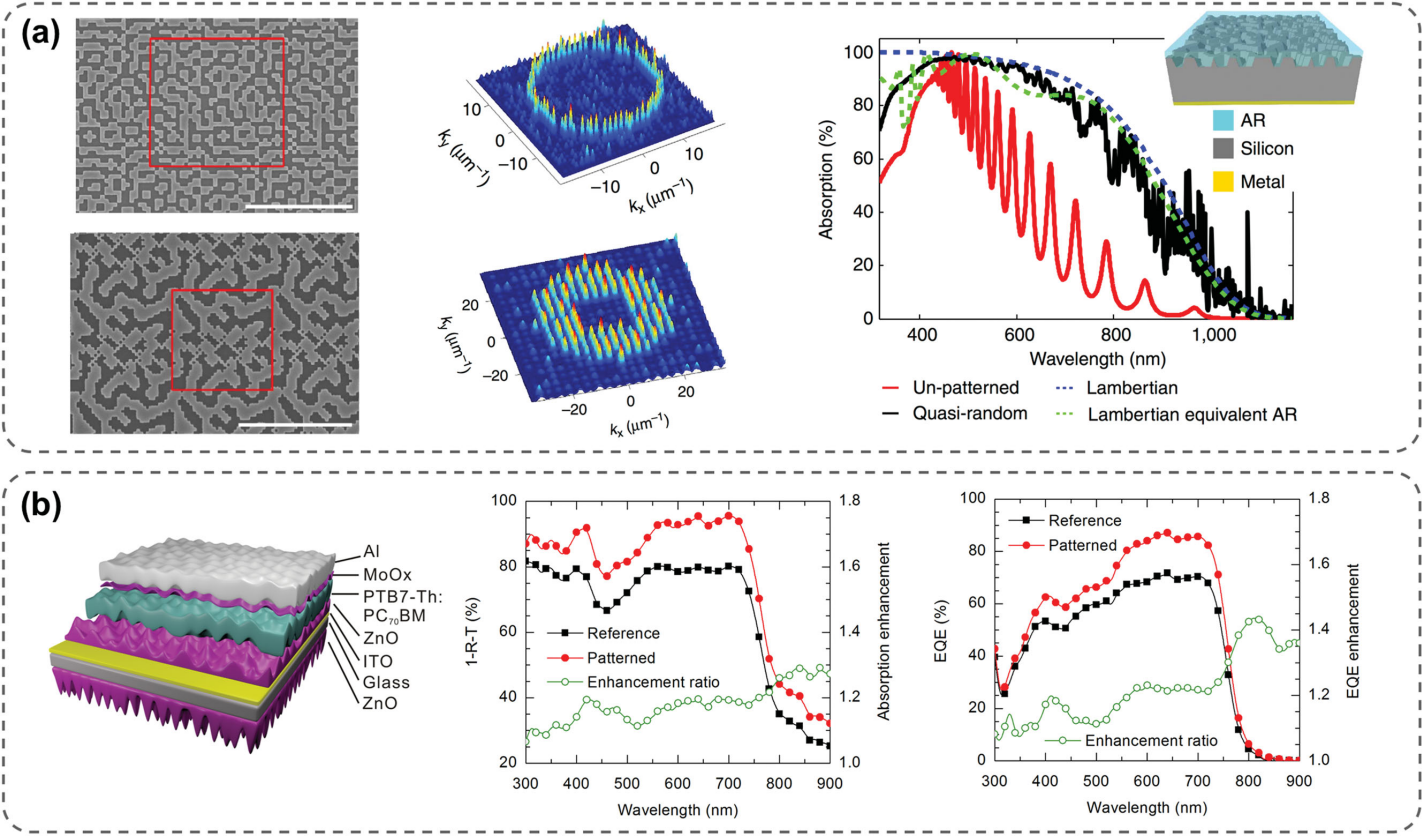
Deterministic aperiodic nanostructures (DANs) for photovoltaics. a) DANs designed with periodic lattice to concentrate the spectral energy with ring‐shaped Fourier spectrumfor light extraction (top left), and with quasi‐random cell to suppress low orders and concentrated the energy into quasi‐guided modes for light trapping (bottom left); broad‐band absorption spectra of nanostructured silicon thin films approaching the Lambertian limit (right). Reproduced with permission.^128^ Copyright 2013, Nature Publishing Group. b) Dual‐sided nanoimprinted DANs in OPVs with improved optical properties. Reproduced with permission.^18^

Instead of the necessity to precisely engineer the nanoscale periodic structures with pre‐specified spacings or at appropriate interfaces, the deterministic aperiodic nanostructures (DANs) suggest an interesting alternative to broadband, wide‐angle light manipulation, which offer unique advantages such as richer Fourier spectra.^224^, ^225^ DANs appear random at first sight, yet they have periodic lattices on an extended unit cell and exhibit spatial frequency properties characteristic of random structures. Recently, Martins et al. developed a novel approach for designing DANs based on binary gratings to control the structures easy to replicate and to tailor towards photovoltaics with broadband light trapping efficiency approaching the theoretical (Lambertian) limit in thin film solar cells (**Figure** **15**a).^128^ In this regard, Chen et al. reported a high efficiency OPVs by tailoring the conventional photoactive layer into a DAN absorber for broadband self‐enhanced light absorption with optimum charge extraction via scalable NIL with uncomplicated fabrication procedures over large areas at low cost (Figure 15b).^18^ Compared to the standard flat architecture, improved light harvesting in OPVs with a nanostructured absorber was realized with an 18% increase in photocurrent and an enhanced PCE exceeding 10%. Based on experimental and theoretical analysis, such a dramatic performance enhancement primarily originated from several collective factors to self‐enhance the light absorption, such as the broadband antireflection, angle‐independent light scattering and polarization‐insensitive SPR effects, as well as minimized recombination probability.

In addition, the implementation of nanostructuring the photoactive layer in OPVs may cause the change of polymer morphology and chain orientation.^226^, ^227^ Aryal et al. reported the use of NIL to fabricate large‐area, high‐density, and ordered nanostructures in conjugated polymer P3HT, and to simultaneously control 3D chain alignment within these P3HT nanostructures.^228^ The chain orientation of imprinted P3HT nanostructures exhibited a strong dependence on their geometry (gratings or pillars). It was claimed that vertical chain alignment observed in both nanogratings and nanopillars indicated strong potential to improve charge transport and optical properties for OPVs. The nanoimprint‐induced molecular orientation for OPVs was also elucidated in other polymer systems in the style of 1D nanograting or 2D nanorods.^229^, ^230^, ^231^ Meanwhile, substrate‐dependent molecular orientation in planar OPVs was observed and showed a great influence on modifying charge transfer process and redistributing optical field, e.g., coupling SPPs.^232^, ^233^, ^234^, ^235^

## 7 Summary and Outlook

We have comprehensively reviewed recent progress in the optical manipulation of light in OPVs at multiple scale from the perspective of plasmonic scattering and photonic resonances. Addressing the critical challenge of insufficient absorption efficiency in ultrathin photoactive films, a variety of light trapping schemes with the implementation of photonic structures have been demonstrated to contribute to improved OPV device performance through increased trapping and enhanced absorption in photoactive layers. It involves a brief comparison of light manipulation between microstructures in ray optics domain and nanostructures of wave optics. A major discussion on light trapping schemes corresponds to the different functional components and their interfaces in thin film OPVs. Antireflection coatings on the external cell surface are implemented at both microscale and nanoscale, without interference with electrical properties and complications in OPV fabrication. Some investigations on altering substrate geometries of OPVs are presented for inspirations of novel, effective cell architectures. Photonic designs on transparent conductors and reflective metal mirrors exhibit optical enhancement by plasmonic scattering and microcavity effects. Optically modified charge extraction and photoactive layers via metallic nanoparticles and photonic structures show strong plasmonic scattering and optical resonant enhancement.

Recent noteworthy achievements in the use of efficient organic absorbers and the implementation of photonic elements are convincing and promising for OPV technology allowing the generation of electrical power at low cost on a very large scale. Taking into account different options and various degrees of freedom in device design, a holistic optical approach to highly efficient OPVs is realizing the best possible light harvesting efficiency without affecting device stability, or compatibility with flexible substrates. We anticipate that the confluence of the plasmonics, nanophotonics, and micro/nano fabrication will achieve new organic‐based photovoltaics. In particular, the promising strategy using non‐fullerene acceptors for low‐cost manufacturing has recently demonstrated high‐performance polymer BHJ OPVs with over 11% PCE and excellent thermal stability.^236^ For small molecule‐based OPVs, a high efficiency of 8.4% has also been achieved by utilizing two fullerene‐free electron‐accepting materials for the long‐range exciton energy transfer.^237^ As the potential alternatives to fullerene molecules and their derivatives, these newly exploited acceptors offer a large absorption overlap with the solar spectrum for sufficient photocurrent generation, together with easily tunable molecular energy levels for efficient carrier extraction. When these highly efficient organic absorbers are integrated with effective light trapping configurations, we can undoubtedly expect an unprecedented level of the efficiency of OPVs.^238^
